# The MUC1–HIF-1α signaling axis regulates pancreatic cancer pathogenesis through polyamine metabolism remodeling

**DOI:** 10.1073/pnas.2315509121

**Published:** 2024-03-28

**Authors:** Divya Murthy, Kuldeep S. Attri, Voddu Suresh, Girish H. Rajacharya, Carlos A. Valenzuela, Ravi Thakur, Junzhang Zhao, Surendra K. Shukla, Nina V. Chaika, Drew LaBreck, Chinthalapally V. Rao, Michael A. Hollingsworth, Kamiya Mehla, Pankaj K. Singh

**Affiliations:** ^a^Eppley Institute for Research in Cancer and Allied Diseases, University of Nebraska Medical Center, Omaha, NE 68198-5950; ^b^Department of Oncology Science, University of Oklahoma Health Sciences Center, Oklahoma City, OK 73104; ^c^Department of Internal Medicine, University of Oklahoma Health Sciences Center, Oklahoma City, OK 73104; ^d^Department of Pathology, University of Oklahoma Health Sciences Center, Oklahoma City, OK 73104; ^e^OU Health Stephenson Cancer Center, University of Oklahoma Health Sciences Center, Oklahoma City, OK 73104

**Keywords:** pancreatic cancer, MUC1, SAT1, hypoxia-inducible factors, polyamine biosynthesis

## Abstract

Dysregulation of polyamine metabolism has been implicated in cancer initiation and progression; however, the mechanism of polyamine dysregulation in cancer is not fully understood. In this study, we investigated the role of MUC1, a mucin protein overexpressed in pancreatic cancer, in regulating polyamine metabolism. Utilizing pancreatic cancer patient data, we noted a positive correlation between MUC1 expression and the expression of key polyamine metabolism pathway genes. Functional studies revealed that knockdown of spermidine/spermine N1-acetyltransferase 1 (*SAT1*), a key enzyme involved in polyamine catabolism, attenuated the oncogenic functions of MUC1, including cell survival and proliferation. We further identified a regulatory axis whereby MUC1 stabilized hypoxia-inducible factor (HIF-1α), leading to increased SAT1 expression, which in turn induced carbon flux into the tricarboxylic acid cycle. MUC1-mediated stabilization of HIF-1α enhanced the promoter occupancy of the latter on *SAT1* promoter and corresponding transcriptional activation of *SAT1*, which could be abrogated by pharmacological inhibition of HIF-1α or CRISPR/Cas9-mediated knockout of *HIF1A*. *MUC1* knockdown caused a significant reduction in the levels of SAT1-generated metabolites, N1-acetylspermidine and N8-acetylspermidine. Given the known role of MUC1 in therapy resistance, we also investigated whether inhibiting SAT1 would enhance the efficacy of FOLFIRINOX chemotherapy. By utilizing organoid and orthotopic pancreatic cancer mouse models, we observed that targeting SAT1 with pentamidine improved the efficacy of FOLFIRINOX, suggesting that the combination may represent a promising therapeutic strategy against pancreatic cancer. This study provides insights into the interplay between MUC1 and polyamine metabolism, offering potential avenues for the development of treatments against pancreatic cancer.

## Significance

Pancreatic cancer is a devastating disease with limited treatment options and poor prognosis. This study uncovers a link between MUC1/hypoxia-inducible factor-1α (HIF-1α) signaling and polyamine metabolism. The study further highlights polyamine metabolism as a key survival pathway in tumor cells and demonstrates the utility of targeting polyamine metabolism in combination with FOLFIRINOX, the current standard of care therapy against advanced metastatic pancreatic ductal adenocarcinoma (PDAC). The combination of FOLFIRINOX with polyamine biosynthesis enzyme spermidine/spermine N1-acetyltransferase 1 (SAT1) inhibitor pentamidine, an Food and Drug Administration (FDA)-approved broad-spectrum anti-infective agent active against several parasitic worms, protozoa, and fungi, showed significantly improved antitumor efficacy compared to FOLFIRINOX alone. Hence, this study can be readily translated into clinic and will form the basis of therapeutic combinations against PDAC.

Pancreatic ductal adenocarcinoma (PDAC) is a highly aggressive malignancy that is often diagnosed at an advanced stage, resulting in a poor prognosis for patients. Despite extensive research efforts, the underlying molecular mechanisms that drive pancreatic cancer progression and metastasis remain poorly understood. Presently, the curative treatment for pancreatic cancer is chemotherapy followed by surgical resection (1). However, due to late diagnosis, early tumor metastasis, and development of chemoresistance, most of the current standard-of-care therapies for pancreatic cancer have limited efficacy (2). Therefore, it is essential to identify new targetable molecular factors that promote pancreatic cancer as the development of effective therapeutic combinations with current standards-of-care therapies is warranted.

MUC1 is a membrane-bound glycoprotein that is aberrantly glycosylated, localized, and expressed in many cancers, including pancreatic cancer (3). The MUC1 oncoprotein has also been identified as a potential therapeutic target for pancreatic cancer due to its frequent overexpression and role in tumor progression, invasion, and metastasis (3–6). MUC1-mediated tumor aggressiveness is in part regulated by MUC1-induced glycolytic and oxidative metabolism in pancreatic and other solid tumors (6–11). Pancreatic cancer is also characterized by a hypoxic microenvironment (12). Hypoxic conditions are known to stabilize hypoxia-inducible factors (HIFs), which act as key drivers of tumor progression and therapeutic resistance (13). We have previously demonstrated that MUC1-mediated stabilization of HIF-1α is critical for metabolic reprogramming in pancreatic cancer cells (6, 11). Hypoxia-mediated metabolic reprogramming in pancreatic cancer involves alterations in several metabolic pathways, including glycolysis, the tricarboxylic acid (TCA) cycle, and fatty acid metabolism. MUC1-mediated anabolic metabolism also contributes to poor response to therapy. MUC1-mediated regulation of nucleotide biosynthesis enhances molecular competition against nucleoside analogs and increases DNA damage repair, imparting poor response to radiotherapy (9, 11).

Another key metabolic pathway dysregulated in pancreatic and other solid tumors is polyamine biosynthesis, which regulates tumor development and progression in PDAC (14, 15). Polyamines are small polycationic molecules that are well known to regulate cellular metabolism, intracellular DNA oxidative damage stress (16, 17), chromatin remodeling, and support cell growth and development (14). Further, polyamines have been shown to promote the progression of pancreatic cancer by activation of oncogene-induced transcriptional regulation and antitumor immunity (14, 18). The polyamine pathway is regulated by several key enzymes, including ornithine decarboxylase (ODC), which is often overexpressed early in pancreatic cancer (19). ODC overexpression leads to increased polyamine synthesis and accumulation, promoting cancer cell growth and survival and increased incidence of solid tumors (20). Recent studies have also highlighted the role of ornithine aminotransferase (OAT1) and spermine synthase (SMS) in inducing tumor progression and migration in pancreatic cancer (14, 21). In accordance with these findings, depletion of polyamines results in diminished tumor growth, and thus, targeting the polyamine pathway has emerged as a promising therapeutic strategy for pancreatic cancer (14). Besides ODC and SMS, Spermidine/Spermine N1-acetyltransferase 1 (SAT1), the polyamine catabolic enzyme that catalyzes the acetylation of spermine and spermidine to N1-acetylspermine and N1-acetylspermidine, respectively, plays an important role in cancer progression. However, the functional implications of SAT1 in different cancers are conflicting. While some studies have highlighted the oncogenic potential of SAT1 in glioblastoma, others have revealed a p53-dependent SAT1-mediated activation of ferroptosis and cell death (22, 23). Furthermore, mechanistic basis of regulation of SAT1 in PDAC is not known.

In this study, we demonstrate that MUC1 oncogene regulates polyamine metabolism in pancreatic cancer cells. The MUC1-mediated regulation of polyamine metabolism is governed by the alterations in SAT1 gene expression. We further demonstrate that MUC1-mediated stabilization of HIF-1α expression is critical for MUC1-induced regulation of SAT1 expression and corresponding activation of polyamine metabolism. We also establish that SAT1 functions as an oncogene through oxidative phosphorylation (OXPHOS) in PDAC, and targeting the *SAT1* gene by genetic deletion or pharmacological inhibition via pentamidine synergizes with the current standard-of-care FOLFIRINOX to provide a therapeutic advantage in mouse models of PDAC.

## Results

### MUC1 Regulates Polyamine Metabolism in Pancreatic Cancer.

**Fig. 1.**
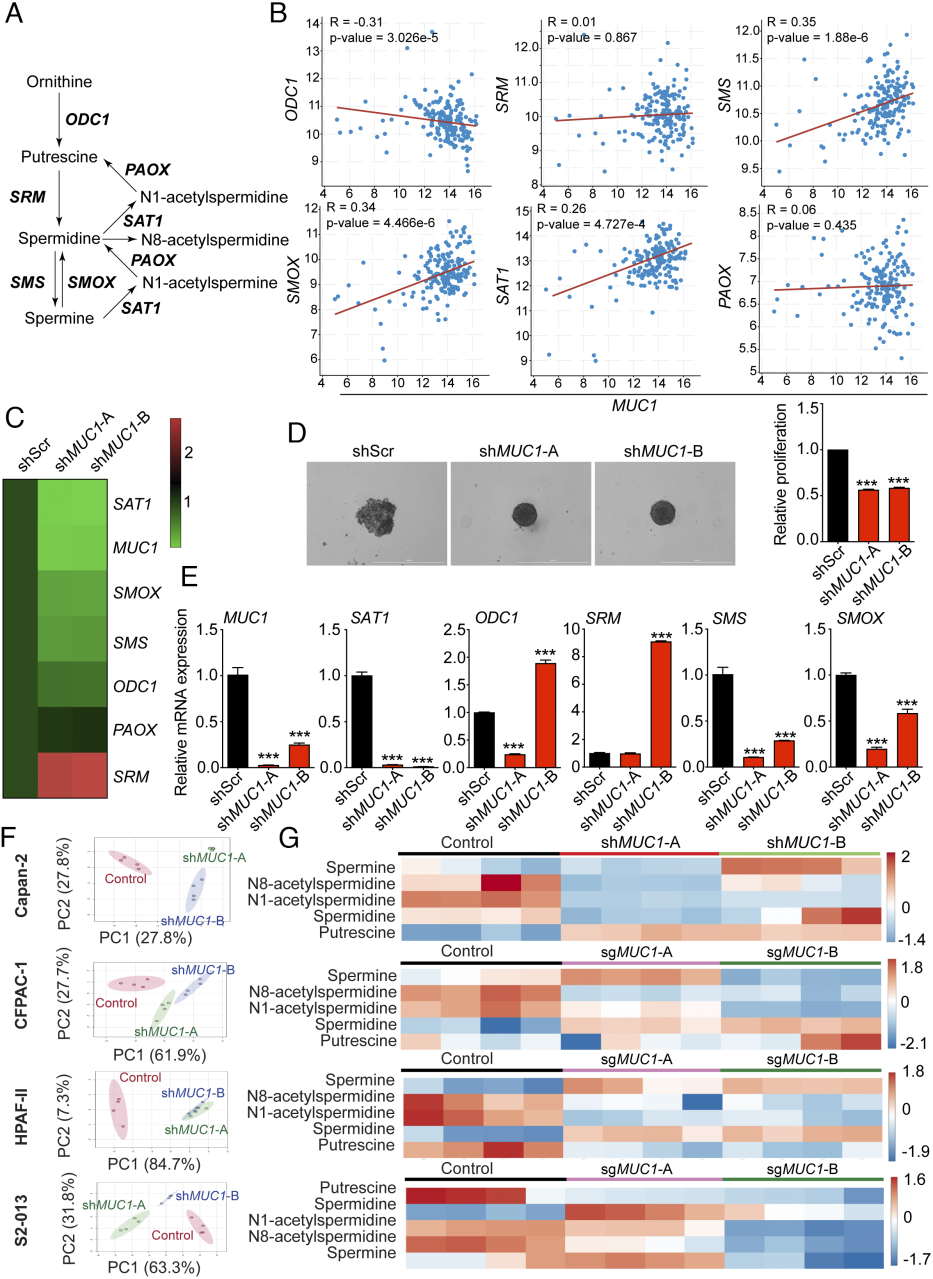
MUC1 positively correlates with polyamine metabolic pathway gene SAT1. (*A*) A schematic illustration of enzyme-coding genes and metabolites of the polyamine biosynthetic pathway. ODC, ornithine decarboxylase 1; PAOX, polyamine oxidase; SAT1, spermidine/spermine N1-acetyltransferase 1; SRM, spermidine synthase; SPS, spermine synthase; SMOX, spermine oxidase. (*B*) Spearman correlation plots showing the correlation of *MUC1* with polyamine metabolic pathway genes (*ODC1*, *SRM*, *SMS*, *SMOX, SAT1*, and *PAOX*). (*C*) The heatmap shows mRNA expression levels of polyamine metabolic pathway genes upon *MUC1* knockdown in Capan-2 cells from RNA-Seq data. (*D*) 3D spheroid assay to assess the growth of Capan-2 cells upon *MUC1* knockdown along with quantitation of relative proliferation. (Scale bar: 1,000 μm.) (*E*) Relative mRNA expression levels of *MUC1* and polyamine metabolic pathway genes in Capan-2 cells upon *MUC1* knock-down. (*F*) The PCA of *MUC1* knockdown Capan-2 and *MUC1* knockout (CFPAC-1, HPAF-II, and S2-013) cells relative to scrambled control cells as determined by LC-MS/MS-based metabolomics. (*G*) Heatmap of polyamine metabolite levels in *MUC1* knockdown Capan-2 and *MUC1* knockout (CFPAC-1, HPAF-II, and S2-013) cells relative to scramble control cells as determined by LC-MS/MS-based metabolomics. The data are represented as mean ± SEM and compared by one-way ANOVA with Tukey’s post hoc test (*D* and *E*). ^∗∗∗^*P* < 0.001.

Polyamine homeostasis is critical for maintaining cellular functions (6, 11). The polyamine pathway metabolites are derived from ornithine metabolism (Fig. 1*A*). Both intracellular and secreted polyamines levels are critical for cellular integrity. We have previously demonstrated that MUC1 is a critical oncogene that regulates pathogenesis and metabolism in PDAC. Furthermore, MUC1 regulates response to chemotherapy and radiotherapy by regulating nucleotide pools (6, 9, 11). Previous studies suggest that nucleotide pools may regulate polyamine metabolism via modulating SAT1 expression (24). Thus, we posited that MUC1 reprograms polyamine metabolism to regulate pathogenesis and therapy response in pancreatic cancer. To investigate this hypothesis, we investigated whether the expression of polyamine pathway genes correlated with *MUC1* mRNA expression in pancreatic cancer patient dataset from the TCGA cohort. While strong positive correlations were noted for mRNA levels of *MUC1* with *SMS*, *SMOX*, and *SAT1*, *ODC1* showed a significant negative correlation (Fig. 1*B*). *SRM* and *POAX* genes were not correlated with *MUC1* (Fig. 1*B*). To test the significance of these correlations, we performed a meta-analysis of polyamine pathway gene regulation in Capan-2 cells upon MUC1 knock-down (25). In line with our hypothesis, we observed a significant reduction in polyamine pathway genes *SAT1, SMS*, and *SMOX*. *SAT1* gene expression was highly decreased upon *MUC1* loss (Fig. 1*C*). To further validate these findings, we utilized CRISPR/Cas9 systems to generate stable knockout of *MUC1* in CFPAC-1, HPAF-II, and S2-013 PDAC cell lines. Consistent with the reported oncogenic role of MUC1, we observed a significant reduction in the growth of the pancreatic cancer cells in 3D-spheroid assays (Fig. 1*D* and *SI Appendix*, Fig. S1*A*). We further observed a significant reduction in the expression levels of *SAT1* and *SMS* genes upon MUC1 loss consistently in all the PDAC cell lines (Fig. 1*E* and *SI Appendix*, Fig. S1*B*). To gain insights into the metabolite-level regulation, we performed LC-MS/MS-based directed metabolomic analysis of polyamine metabolites from scramble control and *MUC1*-depleted PDAC cells. Metabolomic analysis revealed a distinct metabolic profile of *MUC1*-depleted cells as compared to the control cells in the principal component analysis (PCA) plot (Fig. 1*F*). Furthermore, analysis of polyamine metabolites revealed a significant reduction in the levels of SAT1 product metabolites, N1-acetylspermidine and N8-acetylspermidine. Notably, N1-acetylspermidine levels were at least an order of magnitude more abundant than N8-acetylspermidine in all the cell lines and more prominently down-regulated upon SAT1 knockdown (*SI Appendix*, Fig. S1*C*). In addition, an increase in the precursors for the SAT1 enzymatic reaction, spermidine or putrescine was observed (Fig. 1*G* and *SI Appendix*, Fig. S1*C*). These data suggest MUC1 might regulate SAT1 enzyme to modulate polyamine levels. We further noted that MUC1 regulates SAT1 protein levels in Capan-2, CFPAC-1, S2-013 and HPAF-II cells (*SI Appendix*, Fig. S1*D*).

**Fig. 2.**
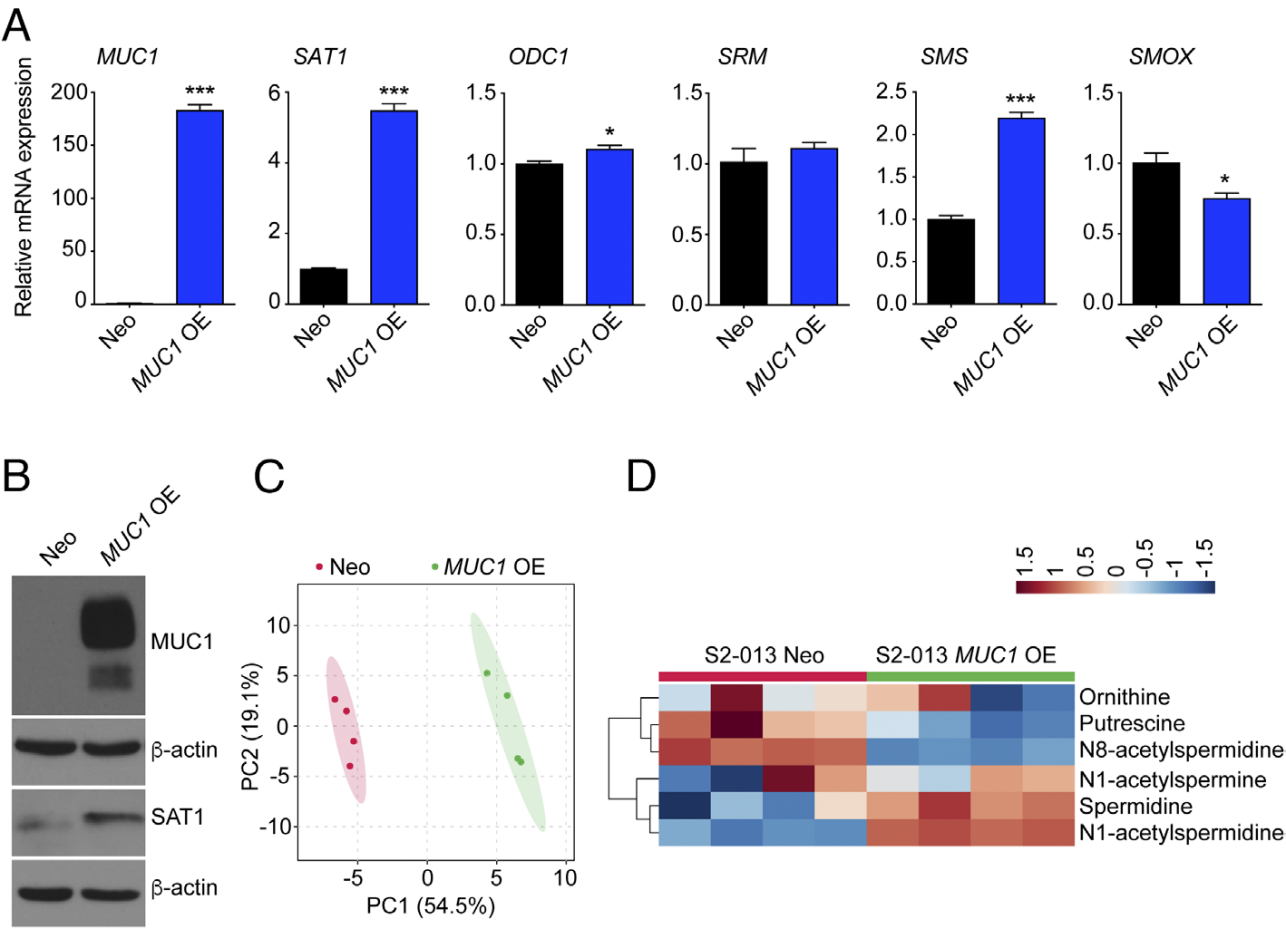
Overexpression of MUC1 increases SAT1 levels and activity in pancreatic cancer cells. (*A*) Relative mRNA expression of *MUC1* and polyamine metabolic pathway genes in S2-013 cells upon *MUC1* overexpression. (*B*) The immunoblots showing the relative levels of MUC1 and SAT1 proteins in control and *MUC1* overexpressing S2-013 cells. (*C*) The PCA of metabolomic profiles of *MUC1* overexpressing S2-013 cells relative to control cells. (*D*) Heatmap of polyamine metabolite levels in *MUC1* overexpressing S2-013 cells relative to control cells. The data are represented as mean ± SEM, and the statistical significance is calculated by Student’s *t* test (*A*). ^∗^*P* < 0.05 and ^∗∗∗^*P* < 0.001.

To further establish the causative link, we overexpressed *MUC1* in S2-013 cells to investigate the link between MUC1 and polyamine metabolic pathway. As expected, we observed an increase in the mRNA levels of *SAT1* and *SMS* genes upon MUC1 overexpression, while we did not observe the expected trend in *SMOX* gene (Fig. 2*A*). Further, an increase in the SAT1 protein expression was noted in MUC1 overexpressing cells (Fig. 2*B*). The metabolomic analysis of polyamine pathway metabolites revealed a distinct clustering between control and MUC1 overexpressing cells (Fig. 2*C*). MUC1 overexpression led to a significant increase in the N1-acetylspermidine levels, whereas a significant reduction was noted in the levels of putrescine (Fig. 2*D* and *SI Appendix*, Fig. S2). Taken together, our data identified MUC1 as a key regulator of polyamine metabolite levels in pancreatic cancer cells through SAT1 enzyme.

### SAT1-Mediates Oncogenic Functions of MUC1 in Pancreatic Cancer Cells.

**Fig. 3.**
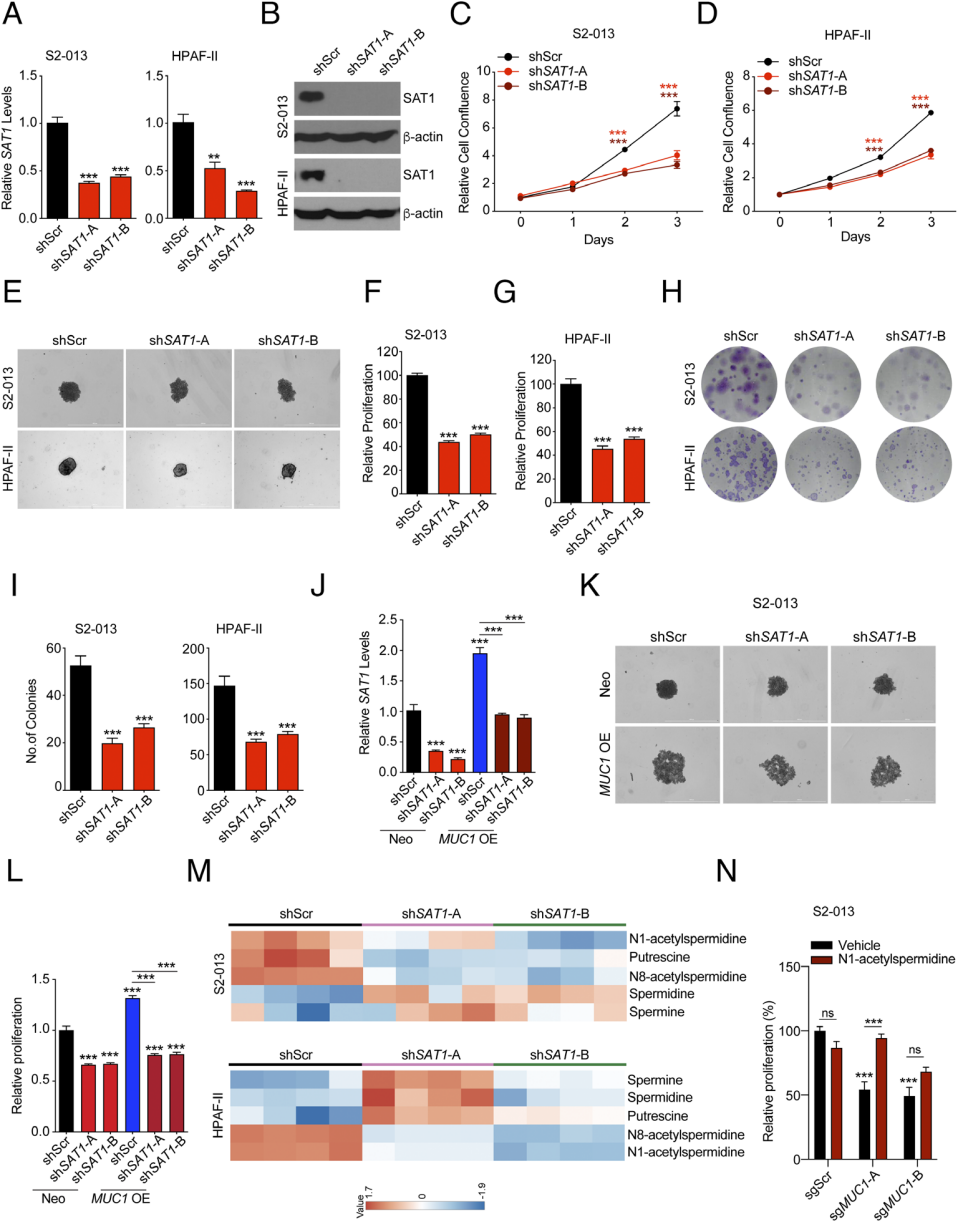
*SAT1* knockdown regulates growth and proliferation of pancreatic cancer cells. (*A*) Relative mRNA expression of *SAT1* in control and *SAT1* knockdown S2-013 and HPAF-II cells. (*B*) SAT1 protein levels in control and *SAT1* knockdown S2-013 and HPAF-II cells by immunoblotting. (*C* and *D*) Relative cell growth as measured by cell confluence of control and *SAT1* knockdown S2-013 (*C*) and HPAF-II (*D*) cells. (*E*) The representative images of 3D spheroid growth assay for control and *SAT1* knockdown S2-013 and HPAF-II cells. (Scale bar: 1,000 μm.) (*F* and *G*) The quantitation of relative proliferation of control and *SAT1* knockdown S2-013 (*F*), and HPAF-II (*G*) cells in 3D spheroid growth assays. (*H*) The representative images of clonogenic assays for control and *SAT1* knockdown S2-013 and HPAF-II cells. (*I*) Quantitative plot indicating the number of colonies from control and *SAT1* knockdown S2-013, and HPAF-II cells. (*J*) Relative *SAT1* mRNA levels in *SAT1* knockdown S2-013 Neo and *MUC1* overexpressing cells compared to the respective scrambled control cells. (*K* and *L*) The representative images (*K*) and quantitation (*L*) of 3D spheroid growth assays for control and *SAT1* knockdown S2-013 Neo and *MUC1* overexpressing cells. (*M*) Heatmap of polyamine metabolite levels in *SAT1* knockdown S2-013 and HPAF-II cells relative to scrambled control cells as determined by LC-MS/MS-based metabolomics. (*N*) Relative proliferation of scrambled control and *MUC1* knockout S2-013 cells upon supplementation with 100 µM N1-acetylspermidine.The data are represented as mean ± SEM and compared by one-way ANOVA with Tukey’s post hoc test (*A*, *C*, *D*, *F*, *G*, *I*, *J, L*, and *N*). ^∗∗^*P* < 0.01 and ^∗∗∗^*P* < 0.001.

To understand the significance of SAT1 in PDAC, the SAT1 gene was knocked-down in two PDAC cell lines, S2-013 and HPAF-II, with high expression of SAT1 (Fig. 3 *A* and *B*). Knockdown of SAT1 resulted in a significant decrease in the survival of the PDAC cell lines (Fig. 3 *C* and *D*). Further, significant reduction in cell growth rates of the PDAC cell lines was observed upon *SAT1* knockdown (Fig. 3 *E*–*G*). Consistent with these findings, knockdown of *SAT1* also abrogated the clonogenic capacity of the PDAC cell lines, establishing the oncogenic function of the *SAT1* gene (Fig. 3 *H* and *I*). We further assessed the contribution of SAT1 in facilitating the oncogenic functions of MUC1 by knocking down *SAT1* in PDAC cells with exogenously overexpressed MUC1 (Fig. 3*J*). Interestingly, diminishing SAT1 gene expression diminished the increased cell growth of MUC1-overexpressing cells (Fig. 3 *K* and *L*). To identify changes in polyamine metabolites associated with SAT1 knockdown, we conducted a metabolomics study. Our analysis revealed a significant decrease in the levels of N1-acetylspermidine in S2-013 and HPAF-II cell lines upon SAT1 knockdown (Fig. 3*M*). Given our hypothesis that MUC1-mediated enhanced pancreatic cancer proliferation is mediated through SAT1, we treated MUC1-depleted cells with N1-acetylspermidine, the product of SAT1. Remarkably, treatment with N1-acetylspermidine rescued the growth defect due to MUC1 depletion (Fig. 3*N*). Interestingly, we observed that N1-acetylspermidine treatment rescued *SAT1* expression in MUC1-depleted HPAF-II and CFPAC-1 cells under hypoxia (*SI Appendix*, Fig. S3 *A* and *B*). These data demonstrate that MUC1 orchestrates its oncogenic function by SAT1-mediated regulation of polyamine homeostasis in PDAC cells.

### MUC1 Regulates SAT1 Expression via HIF-1α Signaling Activation.

**Fig. 4.**
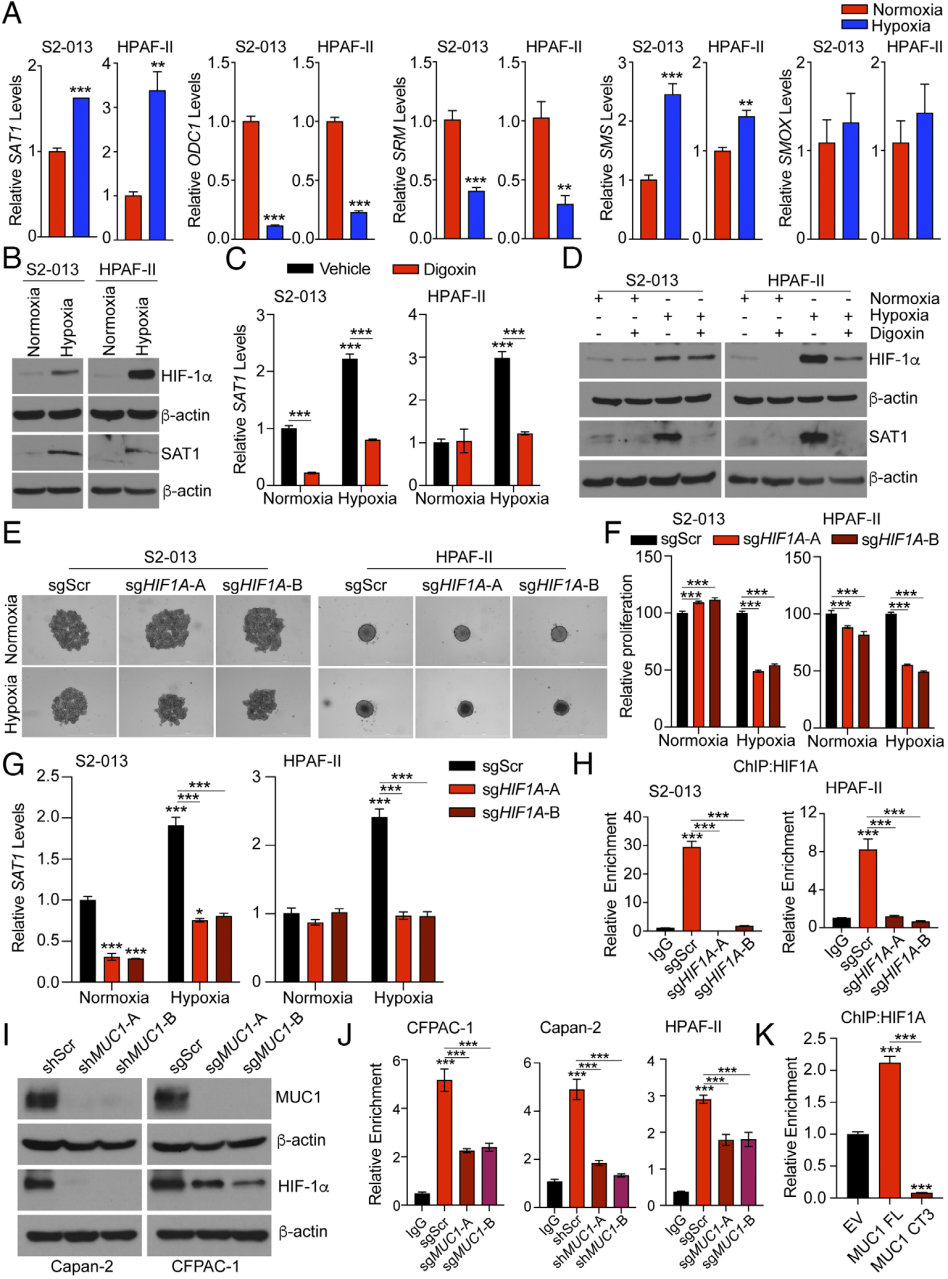
MUC1 upregulates HIF-1α-mediated expression of SAT1 under hypoxia. (*A*) Relative mRNA expression of polyamine pathway genes (*SAT1*, *ODC1*, *SRM*, *SMS*, and *SMOX*) in S2-013 and HPAF-II cells under normoxia and hypoxia. (*B*) HIF-1α and SAT1 protein levels in S2-013 and HPAF-II cells under normoxia and hypoxia by immunoblotting. (*C*) Relative *SAT1* mRNA expression in S2-013 and HPAF-II cells cultured under normoxia and hypoxia upon control or digoxin treatment (100 nM) for 24 h. (*D*) HIF-1α and SAT1 protein levels in S2-013 and HPAF-II cells cultured under normoxia and hypoxia upon control or digoxin treatment for 24 h. (*E* and *F*) The representative images (*E*) and quantitation (*F*) of 3D spheroid growth assays for S2-013 and HPAF-II cells cultured under normoxia and hypoxia upon digoxin treatment for 72 h. (*G*) Relative *SAT1* mRNA expression in S2-013 and HPAF-II cells cultured under normoxia and hypoxia without or with *HIF1A* knockout. (*H*) HIF-1α occupancy, relative to IgG control, on *SAT1* promoter in control and *HIF1A* knockout S2-013 and HPAF-II cells by ChIP PCR. (*I*) MUC1 and HIF-1α protein levels in control and *MUC1* knockdown Capan-2 and CFPAC-1 cells under hypoxia. (*J*) HIF-1α occupancy, relative to IgG control, on *SAT1* promoter in control and *MUC1* depleted CFPAC-1, Capan-2, and HPAF-II cells by ChIP PCR. (*K*) HIF-1α occupancy, relative to IgG control, on *SAT1* promoter in S2-013 cells overexpressing empty vector (EV), full-length MUC1 (FL) or cytoplasmic tail-deleted MUC1 (CT3) protein. The data are represented as mean ± SEM, and the statistical significance is calculated by one-way ANOVA with Tukey’s post hoc test (*C*, *F*, *G*, *H*, *J*, and *K*) or Student’s *t* test (*A*). ^∗^*P* < 0.05, ^∗∗^*P* < 0.01, and ^∗∗∗^*P* < 0.001.

Our previous studies have demonstrated that MUC1 stabilizes HIF-1α transcription factor in pancreatic cancer and activates downstream signaling (6, 11). HIF-1α signaling is critical for the regulation of multiple tumorigenic properties such as cancer cell survival under hypoxic stress, invasion, and chemoresistance. Therefore, we posited that MUC1 regulates *SAT1* expression through HIF-1α signaling. We first assessed the impact of hypoxia on polyamine pathway gene expression. *SAT1* gene expression was induced by hypoxia in each of the four cell lines tested whereas a significant reduction in the expression levels of *ODC1* and *SRM* was observed (Fig. 4*A* and *SI Appendix*, Fig. S4*A*). The hypoxic induction of PDAC cells resulted in increased SAT1 protein levels along with increased HIF-1α levels (Fig. 4*B*). Furthermore, we inhibited HIF-1α using digoxin, a cardiac glycoside that serves as a translational inhibitor of HIF-1α (26). Digoxin treatment abrogated the increase in *SAT1* mRNA levels observed under hypoxia (Fig. 4*C*). Digoxin treatment led to an increase in *ODC1* mRNA levels in both normoxic and hypoxic conditions (*SI Appendix*, Fig. S4 *B* and *C*). No consistent pattern of regulation was observed in the regulation of other polyamine pathway genes (*SI Appendix*, Fig. S4 *B* and *C*). HIF-1α-mediated regulation of SAT1 was also confirmed at the protein level by immunoblotting (Fig. 4*D*). To validate the results from the pharmacological inhibition of HIF-1α, we generated a genetic knockout of HIF-1α-coding gene (*HIF1A*) in S2-013 and HPAF-II pancreatic cancer cells by utilizing CRISPR/Cas9 technology (*SI Appendix*, Fig. S4 *D* and *E*). Deletion of HIF-1α resulted in significantly reduced growth of pancreatic cancer cells under hypoxia (Fig. 4 *E* and *F*). Consistent with the data from the pharmacological inhibition of HIF-1α, we observed that *HIF1A* knockout reduced the expression of the *SAT1* gene under hypoxic conditions in both S2-013 and HPAF-II PDAC cell lines (Fig. 4*G*). Interestingly, ODC1 levels were increased upon *HIF1A* knockout, and no specific pattern of regulation could be established for other polyamine pathway genes (*SI Appendix*, Fig. S4 *F* and *G*). We then investigated whether HIF-1α transcription factor occupied the promoter region of the *SAT1* gene. Along these lines, we observed the presence of HIF binding motif (hypoxia response element; HRE) in the promoter region of the SAT1 gene using JASPAR and PROMO ALLGEN (*SI Appendix*, Fig. S4*H*). As expected, an increased binding of HIF-1α was observed in the *SAT1* promoter region exclusively in scramble control cells, and the binding was abolished in the *HIF1A* knockout S2-013 and HPAF-II cells (Fig. 4*H*). To identify whether HIF-1α mediates the regulatory mechanism governing MUC1-mediated regulation of *SAT1*, we assessed the expression of HIF-1α in *MUC1*-depleted pancreatic cancer cell lines. As predicted, the depletion of *MUC1* resulted in markedly reduced expression of HIF-1α possibly through reduced HIF-1α stabilization as reported in our earlier studies (6) (Fig. 4*I*). We further dissected the molecular complex facilitating the MUC1-mediated *SAT1* expression by chromatin immunoprecipitation (ChIP) studies. While deletion of MUC1 in PDAC cell lines led to a decrease in the occupancy of HIF-1α on the SAT1 gene promoter (Fig. 4*J*), overexpression of wild-type MUC1 (MUC1FL) in S2-013 cells resulted in increased occupancy of HIF-1α on the *SAT1* gene promoter. Interestingly, exogenous expression of cytoplasmic tail-deleted MUC1 in S2013 cells [MUC1 CT3 (27)] significantly reduced the binding of HIF-1α on *SAT1* promoter and decreased expression suggesting that the molecular complex comprising of HIF-1α and functional MUC1 is pertinent for the regulation of *SAT1* gene expression in PDAC cells (Fig. 4*K* and *SI Appendix*, Fig. S4*I*). Taken together, our data suggests that MUC1 regulates *SAT1* expression through HIF-1α signaling in hypoxic microenvironments.

We also investigated the potential reciprocal regulation of HIF-1α by SAT1, exploring the possible regulatory mechanism for the modulation of HIF-1α by the *SAT1* gene. Western blot analysis of HIF-1α expression in *SAT1* knockdown cells under hypoxia revealed a decrease in the expression of HIF-1α (*SI Appendix*, Fig. S4*J*). Additionally, the expression of some of the HIF-1α-regulated genes was decreased in *SAT1* knockdown cells under both normoxic and hypoxic conditions (*SI Appendix*, Fig. S4*K*). These findings strongly suggest that SAT1 may play a role in the regulation of HIF-1α and its associated pathways.

### SAT1 Reprograms Pancreatic Cancer Metabolism to OXPHOS.

**Fig. 5.**
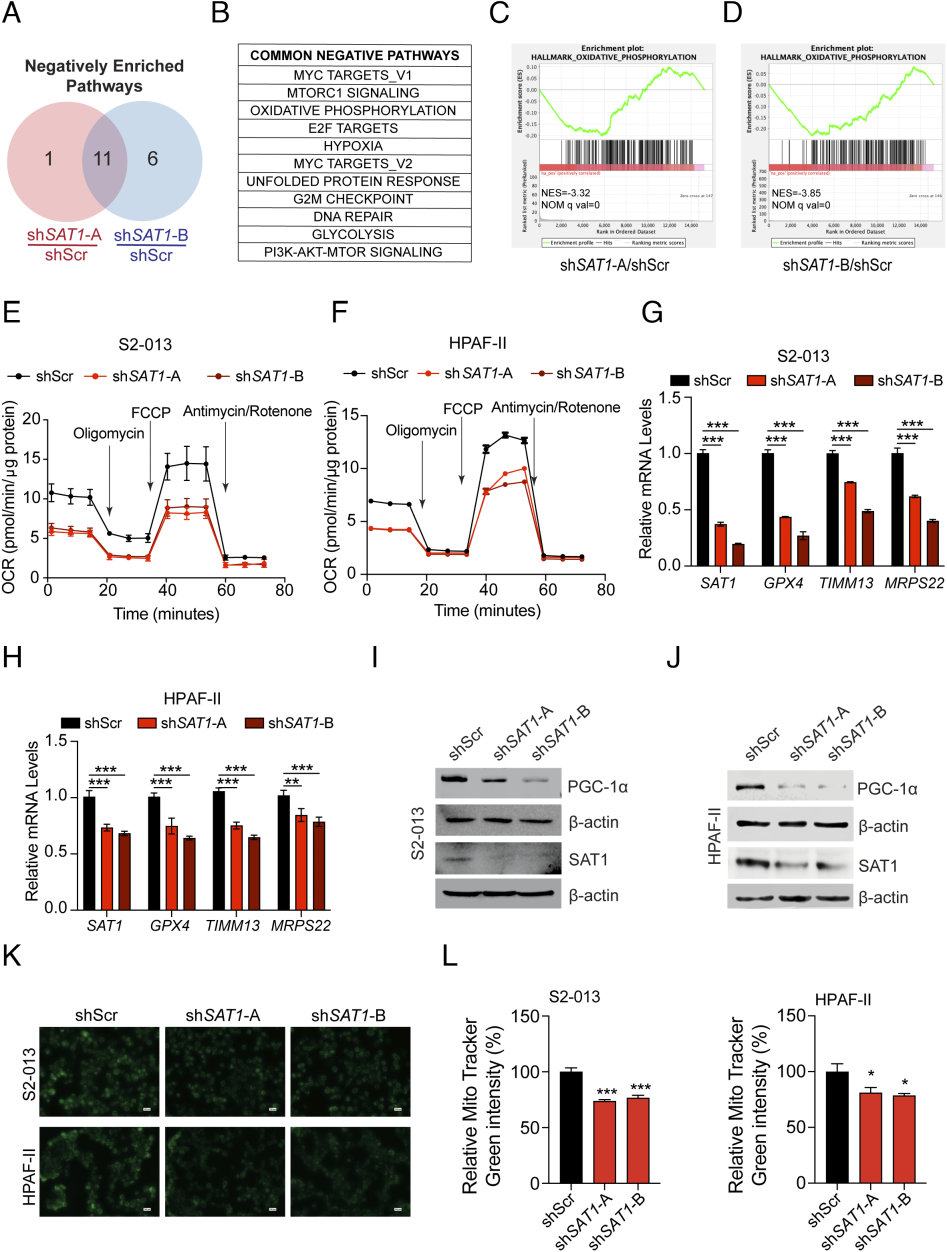
SAT1 reprograms mitochondrial metabolism in pancreatic cancer cells. (*A*) Venn diagram of negatively enriched pathways from GSEA of sh*SAT1*-A and sh*SAT1*-B cells compared to shScr S2-013 cells. The pathways with an NES greater than 2 and q-value less than 0.05 were considered for analysis. (*B*) List of common negative enriched pathways in *SAT1* knockdown cells compared to scramble control cells. (*C* and *D*) GSEA plot of OXPHOS in sh*SAT1*-A (*C*) and sh*SAT1*-B (*D*) S2-013 cells compared to shScr controls. (*E* and *F*) Seahorse-based metabolic flux analysis of OCR in control and *SAT1* knockdown S2-013 (*E*) and HPAF-II (*F*) cells. (*G* and *H*) Relative mRNA expression of *SAT1, GPX4, TIMM13,* and *MRPS22* genes in scrambled control and *SAT1* knockdown S2-013 (*G*) and HPAF-II (*H*) cells. (*I* and *J*) Relative levels of PGC-1α and SAT1 proteins in scrambled control and SAT1 knockdown in S2-013 (*I*) and HPAF-II (*J*) cells. (*K* and *L*) Representative images (*K*) of MitoTracker green-stained scrambled control and *SAT1* knockdown S2-013 and HPAF-II cells along with quantification of dye staining (*L*). (Scale bar: 100 μm.) The data are represented as mean ± SEM, and the statistical significance is calculated by one-way ANOVA with Tukey’s post hoc test (*G*, *H*, and *L*). ^∗^*P* < 0.05, ^∗∗^*P* < 0.01, and ^∗∗∗^*P* < 0.001.

To gain insights into the mechanistic regulation by the *SAT1* gene, we performed RNA-Seq analysis of scramble control and *SAT1* knockdown S2-013 cells. GSEA pathway enrichment analysis revealed 11 negatively enriched pathways with *SAT1* loss (Fig. 5 *A* and *B*). Myc target genes, OXPHOS, hypoxia, and G2M checkpoint were key significantly regulated pathways (Fig. 5 *A* and *B* and *SI Appendix*, Fig. S5 *A*–*D*). Earlier studies have noted that OXPHOS is critical for supporting the growth of cancer cells (28). Our transcriptomic data also suggests regulation of OXPHOS by the *SAT1* gene (Fig. 5 *C* and *D*). To validate our findings, we performed seahorse-based metabolic flux analysis in control and *SAT1* knockdown cells. Knocking down the *SAT1* gene in S2-013 and HPAF-II cells led to an overall reduction in OXPHOS as evidenced by decreased oxygen consumption rate (OCR) (Fig. 5 *E* and *F*). Given that SAT1 knockdown led to a reduction in the OCR, we examined the impact of SAT1 depletion on mitochondrial genes. In alignment with the transcriptomics analysis and metabolic flux assessment, SAT1 knockdown resulted in a decreased expression of mitochondrial genes (Fig. 5 *G* and *H* and *SI Appendix*, Fig. S5 *E* and *F*). Furthermore, we examined the kinetics of [U-^13^C]glucose labeling of TCA metabolites in scrambled and *SAT1* knockdown cells. We observed slower glucose flux into TCA metabolites in *SAT1* knockdown cells compared to scrambled cells (*SI Appendix*, Fig. S5*G*). Additionally, we explored the regulation of PGC-1α, a master regulator of mitochondrial biogenesis. Notably, consistent with the observed reduction in mitochondrial mass upon *SAT1* knockdown, the expression of PGC-1α was diminished upon SAT1 knockdown in PDAC cells (Fig. 5 *I*–*L*). Collectively, these findings indicate that SAT1 modulates mitochondrial homeostasis, thereby regulating mitochondrial bioenergetics in pancreatic cancer cells.

### The MUC1–SAT1 Axis Regulates Pancreatic Cell Growth through OXPHOS.

**Fig. 6.**
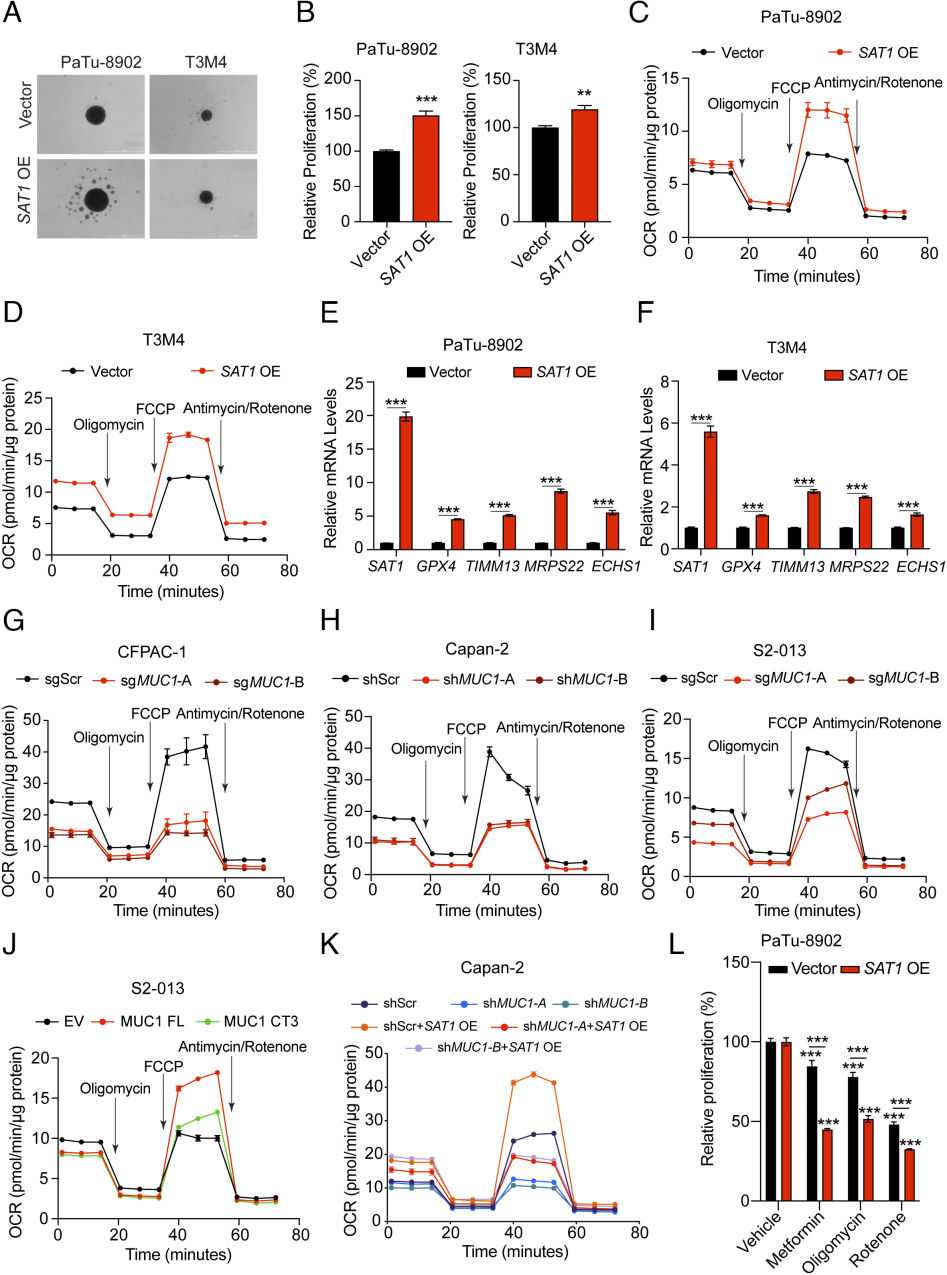
MUC1–SAT1 axis–mediated regulation of OXPHOS is critical for pancreatic cancer cell growth. (*A* and *B*) Representative images (*A*) of 3D spheroid growth assay of PaTu-8902 and T3M4 cells upon overexpression of the *SAT1* gene along with quantification of relative proliferation (*B*). (Scale bar: 1,000 μm.) (*C* and *D*) Seahorse-based metabolic flux analysis of OCR in vector control and *SAT1* overexpressing PaTu-8902 (*C*) and T3M4 (*D*) cells. (*E* and *F*) Relative mRNA expression of *SAT1, GPX4, TIMM13, MRPS22,* and *ECHS1* genes in vector control and *SAT1* overexpressing PaTu-8902 (*E*) and T3M4 (*F*) cells. (*G*–*I*) Seahorse-based metabolic flux analysis of OCR in scramble control and MUC1 knockout CFPAC-1 (*G*), MUC1 knockdown Capan-2 (*H*), and *MUC1* knockout S2-013 (*I*) cells. (*J*) Seahorse-based metabolic flux analysis of OCR in S2-013 cells overexpressing empty vector (EV), FLAG epitope-tagged full-length *MUC1* (FL) or cytoplasmic tail-deleted MUC1 (CT3) protein. (*K*) Seahorse-based metabolic flux analysis of OCR in scramble or *MUC1* knockdown Capan-2 cells upon overexpression of the *SAT1* gene. (*L*) Relative proliferation of vector control and *SAT1* overexpressing PaTu-8902 cells upon treatment with OXPHOS inhibitors (5 mM metformin, 3 nM oligomycin, and 10 μM rotenone). Each treatment group was normalized to the respective vehicle-treated group. Bar charts are represented as mean ± SEM compared by and unpaired Student’s *t* test (*B*, *E*, and *F*) and one-way ANOVA with Tukey’s post hoc test (*L*). ^∗^*P* < 0.05, ^∗∗^*P* < 0.01, and ^∗∗∗^*P* < 0.001.

To establish the oncogenic function of *SAT1*, we overexpressed SAT1 in T3M4 and PaTu-8902 pancreatic cancer cell lines, with lower basal *SAT1* expression. The overexpression of *SAT1* in these cell lines resulted in increased growth in 3D spheroids and an enhanced OCR, signifying improved OXPHOS (Fig. 6 *A*–*D*). Moreover, the overexpression of *SAT1* also led to an increased expression of mitochondrial genes (Fig. 6 *E* and *F*). In line with the *SAT1* overexpression results, treatment with N1-acetylspermidine also led to increased expression of mitochondrial genes (*SI Appendix*, Fig. S6*A*).

We have previously demonstrated that the depletion of MUC1 decreases glycolysis (11). In line with our previous findings, a decrease in extracellular acidification rate (ECAR) was observed in PDAC cell lines upon *MUC1* depletion (*SI Appendix*, Fig. S6 *B*–*D*). Since we demonstrated that MUC1 regulates *SAT1* expression, we therefore investigated the impact of MUC1 depletion on OXPHOS. Interestingly, we observed a significant reduction in OCR in multiple PDAC cell lines upon *MUC1* loss (Fig. 6 *G*–*I*). Our data here suggests loss of MUC1 reprograms both glycolysis and OXPHOS pathways. To further establish the significance of OXPHOS regulation, we examined the kinetics of [U-^13^C]glucose labeling of TCA metabolites in scrambled and *MUC1* knockout cells. Consistent with the previous data, we observed reduced glucose flux into TCA metabolites in *MUC1* knockout cells compared to scrambled cells (*SI Appendix*, Fig. S6*E*). In addition, the overexpression of MUC1FL in S2-013 cells led to an increase in OCR (Fig. 6*J*). However, the exogenous expression of MUC1-CT3 was unable to enhance the OCR and was comparable to the cells expressing the vector alone (Fig. 6*J*). Furthermore, to validate the MUC1–SAT1 metabolic axis in the regulation of PDAC proliferation, we conducted rescue experiments by overexpressing *SAT1* in *MUC1* knockdown cells. As expected, *SAT1* overexpression resulted in the rescue of OCR in *MUC1*-depleted cells (Fig. 6*K* and *SI Appendix*, Fig. S6*F*). To ascertain whether enhanced OXPHOS specifically contributes to the growth-promoting effects of SAT1 in PDAC, we performed 3D spheroid-based growth assays in SAT1-overexpressing cells in the presence of OXPHOS inhibitors. In concordance with OCR analysis, the overexpression of SAT1 resulted in increased growth of PDAC cells which was abrogated upon treatment with OXPHOS inhibitors (Fig. 6*L* and *SI Appendix*, Fig. S6*G*). Taken together, our results here suggest that the MUC1–SAT1 axis rewires OXPHOS to regulate cellular proliferation in PDAC cells.

### FOLFIRINOX and Pentamidine Synergistically Regulate Pancreatic Tumor Growth.

**Fig. 7.**
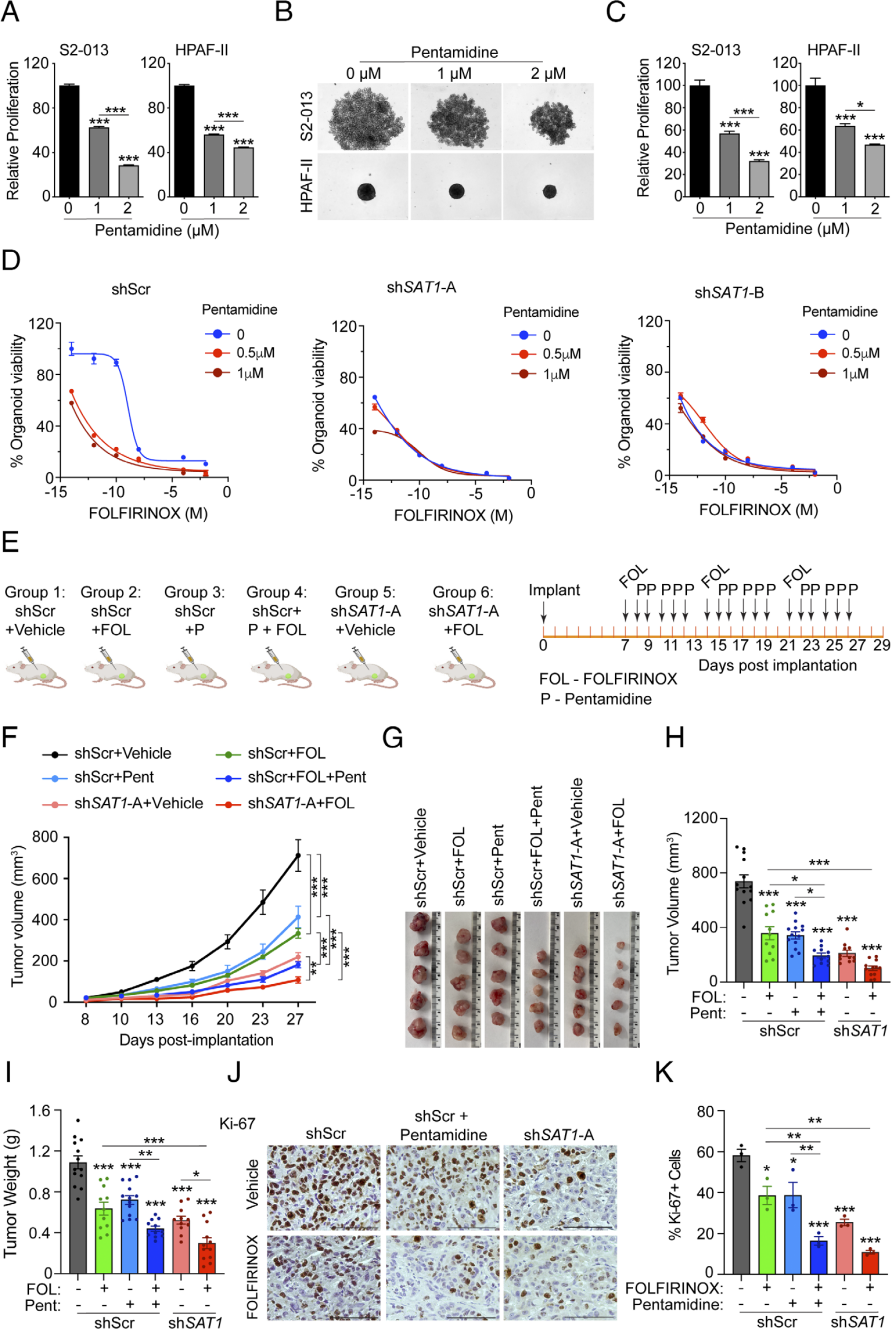
Targeting SAT1 enhances FOLFIRINOX efficacy against PDAC in vivo. (*A*) Relative proliferation of S2-013 and HPAF-II cells upon treatment with pentamidine for 72 h. (*B* and *C*) The representative images (*B*) and quantitation (*C*) of 3D spheroid growth assay in S2-013, and HPAF-II cells upon treatment with pentamidine for 72 h. (*D*) Dose–response curves of scrambled control or SAT1 knockdown PA901 organoids-treated with 0.5 or 1 μM pentamidine in combination with multiple doses of FOLFIRINOX. (*E*) The schematic of pentamidine (P) and/or FOLFIRINOX (F) dosing in athymic nude mice orthotopically implanted with shScr or sh*SAT1* S2-013 cells. (*F*–*I*) Tumor growth kinetics in athymic nude mice implanted with shScr or sh*SAT1*-A S2-013 cells upon treatment with FOLFIRINOX (FOL) without or with pentamidine (Pent), and corresponding postnecropsy representative tumor images (*G*), tumor volumes (*H*), and tumor weights (*I*). (*J* and *K*) Representative IHC staining (*J*) and quantitation in three different fields from three tumor sections of each group (*K*) for Ki-67 in the formalin-fixed tumor sections from athymic nude mice implanted with shScr or sh*SAT1*-A S2-013 cells treated with FOLFIRINOX without or with pentamidine. (Scale bar, 100 μm.) The data are represented as mean ± SEM, and the statistical significance is calculated by one-way ANOVA with Tukey’s post hoc test (*A*, *C*, *H*, *I*, and *K*) or two-way ANOVA with Tukey’s test (*F*). ^∗^*P* < 0.05, ^∗∗^*P* < 0.01, and ^∗∗∗^*P* < 0.001.

To investigate the therapeutic potential of SAT1-mediated molecular processes in pancreatic cancer, we utilized pentamidine, a pharmacological inhibitor of SAT1. Pentamidine treatment of PDAC cells led to a significant reduction in the growth of pancreatic cancer cells in both 2D growth assays and tumor spheroid assays (Fig. 7 *A*–*C* and *SI Appendix*, Fig. S7 *A*–*C*). Next, we conducted an in vitro experiment using a tumor-derived organoid model. Specifically, we induced *SAT1* knockdown in the PA907 organoid line, which resulted in the reduced growth of the organoids (Fig. 7*D*). To assess the specificity of pentamidine toward SAT1, we treated scrambled and *SAT1*-depleted tumor organoids with increasing concentrations of pentamidine in the presence of different doses of FOLFIRINOX. Interestingly, while *SAT1* knockdown sensitized the organoids to FOLFIRINOX, it also precluded any additional effects of pentamidine on organoid growth, suggesting on-target effect of pentamidine for growth inhibition in the model (Fig. 7*D*). Subsequently, we tested whether pentamidine can be combined with FOLFIRINOX, the standard of care therapy for pancreatic cancer patients to develop an effective and potent therapeutic regimen. Coimplantation of human pancreatic stellate (HPS) cells with pancreatic cancer cells in mice mimics tumor growth kinetics and therapy response in patients by creating hypoxic microenvironments (29). We coimplanted HPS: tumor cells in 1:1 ratio in athymic nude mice for chemotherapy response studies (Fig. 7*E*). To rule out potential off-target effects of pentamidine contributing to the improved therapy response, we included additional cohorts of mice coimplanted with sh*SAT1* and HPS cells and treated with control or FOLFIRINOX therapy. The combined treatment of FOLFIRINOX with pentamidine showed a significant decrease in the tumor growth kinetics, compared to either treatment alone (Fig. 7*F*). Furthermore, *SAT1* knockdown caused a significant decrease in pancreatic tumor burden, and treatment with FOLFIRINOX further reduced the tumor growth kinetics (Fig. 7*F*). A significant reduction in tumor volume and weight parameters was observed upon necropsy in mice receiving a combined treatment of FOLFIRINOX and pentamidine compared to individual treatment groups (Fig. 7 *G*–*I*). Furthermore, a significant reduction was observed in tumor volume and tumor weight in FOLFIRINOX-treated *SAT1* knockdown cells compared to FOLFIRINOX treatment alone or *SAT1* knockdown alone cohorts (Fig. 7 *G*–*I*). We also observed no significant reduction in the body weight of combination therapy mice compared to individual treatment groups, suggesting the therapy is well tolerated (*SI Appendix*, Fig. S7*D*). Consistent with the tumor growth data, we observed a significant reduction in Ki-67-positive proliferating tumor cells and an increase in cleaved caspase-3 staining in tumor sections from combined therapy group, as compared to either treatment alone (Fig. 7 *J* and *K* and *SI Appendix*, Fig. S7*E*). A similar reduction in the number of Ki-67-positive cells and a corresponding increase in cleaved caspase-3 staining was observed in FOLFIRINOX-treated *SAT1* knockdown groups, as compared to *SAT1* knockdown or FOLFIRINOX-treated mice groups (Fig. 7 *J* and *K* and *SI Appendix*, Fig. S7*E*). In conclusion, our preclinical study suggests an efficacious combination therapy of FOLFIRINOX and pentamidine for pancreatic cancer.

## Discussion

The dysregulation of polyamine metabolism has been implicated in various solid tumors, including PDAC (14). In the present study, we have identified a regulatory mechanism of the polyamine biosynthetic pathway via the MUC1–HIF-1α-SAT1 signaling axis. Our study demonstrates a positive correlation between MUC1 expression and the expression of a key polyamine metabolic pathway gene, SAT1, in PDAC patients. Mechanistically, we observed that MUC1-mediated stabilization of HIF-1α induces SAT1, which switches the metabolic status of the PDAC cells toward OXPHOS. In addition, the depletion of *SAT1* sensitized PDAC tumors to the chemotherapeutic agent, FOLFIRINOX.

MUC1 is a transmembrane protein with well-known oncogenic functions in PDAC and is known to impart transcriptional alterations that lead to metabolic reprogramming in cancer cells (6, 10, 11). While our previous studies have demonstrated the regulation of glycolytic metabolism in pancreatic cancer or glutamine metabolism in triple-negative breast cancer, our results from this study shed light on the functional implications of the MUC1 oncoprotein in pancreatic cancer progression through the regulation of polyamine biosynthesis (6, 8). SAT1, a key enzyme involved in polyamine catabolism, regulates tumor growth and survival in multiple cancer types. While some studies have reported low expression of SAT1 in human cancer patient specimens, others, in particular glioblastoma and low-grade gliomas, report an oncogenic function of SAT1 (22, 23, 30). Our current study demonstrates that *SAT1* knockdown diminishes the oncogenic functions of MUC1, including cell survival, proliferation, and clonogenic capacity, underscoring the significance of the MUC1–SAT1 axis in PDAC.

Furthermore, we revealed a mechanism by which MUC1 regulates SAT1 expression through the stabilization of HIF-1α, a key transcription factor involved in hypoxic response. Under hypoxic conditions, HIF-1α can regulate the polyamine biosynthetic and homeostatic pathways in cancer cells and other cellular systems such as retinal glial cells (31, 32). The effects of HIF-1α are primarily mediated by increased stabilization, and attenuated ubiquitination and proteasomal degradation by the ubiquitin-ligase complex (33). Stabilization and activation of HIF-1α by MUC1 have been well established in the development of chemoresistance in PDAC cells (6, 11). In contrast, SAT1 binding to HIF-1α promotes ubiquitination and further degradation of the latter (34). However, the regulation of SAT1 by hypoxia and HIF-1α was not studied. Here, we demonstrate that inhibition or knockout of HIF-1α reduced SAT1 expression and impaired cell proliferation under hypoxic conditions suggesting a cross-regulation between HIF-1α and SAT1 to maintain polyamine homeostasis in PDAC cells. Interestingly, we observed an opposite correlation between HIF-1α and ODC1 unlike previous reports (31). These findings demonstrate that HIF-1α is a critical regulator of polyamine metabolism and PDAC progression, further supporting the functional relevance of the MUC1–HIF-1α–SAT1 signaling axis in PDAC.

The role of polyamines in the immune system and their relationship with the gut or intratumoral microbiome is an emerging area of research (35). Polyamines play a crucial role in regulating immune responses by modulating the activity of immune cells such as T cells, B cells, natural killer cells, and macrophages (36). In addition, polyamines can influence the composition and activity of the gut microbiota, shaping the microbial community structure and function. Conversely, the gut microbiome also plays a crucial role in polyamine metabolism by synthesizing polyamines that contribute to the overall polyamine pool in the body (37). Therefore, targeting polyamine metabolism or the gut microbiome–mediated polyamine biosynthesis could be a potential therapeutic strategy for treating pancreatic cancer. However, utilization of immunocompromised mouse model in our in vivo studies hindered assessment of how the immune system or gut microbiome regulates polyamine-mediated effects in pancreatic cancer. Additional studies will be needed to investigate the intricate interactions among polyamines, immune system, and the microbiome, to provide perspectives beyond polyamines’ cell-autonomous effects.

Previous studies have demonstrated the potential of targeting polyamine metabolism in cancer prevention, including pancreatic cancer (19). Targeting polyamine biosynthesis by inhibiting ODC1 using difluoromethylornithine has had limited clinical success for solid tumors (38, 39). Our study demonstrated that pharmacological inhibition of SAT1 using pentamidine synergistically enhanced the antitumor effects of FOLFIRINOX, the current standard-of-care therapy for PDAC (40), in an orthotopic mouse model of PDAC. Importantly, our study suggests that targeting SAT1 alone or in combination with existing therapies could represent a promising therapeutic strategy for PDAC patients. These findings highlight the potential of SAT1-targeted therapies to improve treatment outcomes and overcome chemotherapy resistance in PDAC patients.

In conclusion, our study provides insights into the role of the MUC1–HIF-1α–SAT1 signaling axis in regulating polyamine metabolism and promoting PDAC tumorigenesis. Targeting SAT1, either alone or in combination with existing therapies, holds promise as a therapeutic approach for PDAC patients. These studies are expected to facilitate future research to evaluate the efficacy of SAT1-targeted therapies in clinical settings.

## Materials and Methods

### Cell Culture, Cell Lines, and Chemicals.

CFPAC-1, Capan2, and HPAF-II pancreatic cancer cells and HEK293T cells were obtained from the American Type Culture Collection (Rockville, MD). S2-013 is a cloned subline of a human pancreatic tumor cell line (SUIT-2) derived from liver metastasis. S2-013 parental cell line, transfectants of the S2-013 cells [S2-013.Neo, S2-013.MUC1F, and S2-013.MUC1F.CT3], T3M4, and PaTu-8902 were provided by Michael A. Hollingsworth (Eppley Institute, UNMC, Omaha, NE) (41, 42). HPS cells were provided by Rosa F. Hwang (MD Anderson Cancer Center, Houston, TX) (29). The cells were cultured in Dulbecco’s Modified Eagle’s Medium (DMEM) supplemented with 10% fetal bovine serum and 1:100 antibiotic-antimycotic (Gibco). The cell lines were routinely tested for *mycoplasma* contamination and confirmed by STR profiling at the University of Arizona Genetics Core. Pentamidine (Cayman Chemical, 20679), 2-deoxyglucose (Sigma-Aldrich, D8375), oligomycin (Sigma-Aldrich, O4876), FCCP (Sigma-Aldrich, C2920), Rotenone (Sigma-Aldrich, R8875), Antimycin A (Sigma-Aldrich, A8674), Turbofect transfection reagent (Thermo Fisher Scientific, R0532), Protein G Magnetic Beads (Thermo Fisher Scientific, 10004D), PowerUP SYBR Green Mastermix (Applied Biosystems, A25742), and 3-[4,5-dimethylthiazol-2-yl]-2,5-diphenyltetrazolium bromide (MTT) (Sigma Aldrich, M2128), and HA-magnetic beads (Thermo Fisher Scientific, 88836) were utilized as indicated.

### Transcriptome Sequencing Analysis.

Total RNA was isolated from scrambled control and *SAT1* knockdown (sh*SAT1*-A and sh*SAT1*-B) cells using the Qiagen RNAeasy mini kit (Germantown, MD), and library was prepared for sequencing. The library quality was evaluated using an Agilent 2100 Bioanalyzer before sequencing through an Illumina system. TopHat2 was used for alignment and differential expression was done through DESeq2 R package. For defining the differentially expressed genes, we utilized a fold change ≥1.5 for up- or downregulation. The log ranked file generated for differentially expressed between different groups was processed through GSEA2 v2.2.3 with 1,000 permutations in the classic scoring scheme using the c2.cp.kegg.v6.2.symbols.gmt and c2.tft.v6.1.symbols.gmt geneset database from the Broad Institute. Heatmaps were generated using GraphPad Prism 8.

### Tumor Spheroid Assay.

2 × 10^3^ cells were seeded per well in a round bottom low attachment 96-well plates (Corning Inc., Corning, NY) in DMEM/F12 media supplemented with 1% B27, 20 ng/mL EGF and FGF. Plates were centrifuged at 900 g and treated with the respective drugs after 48 h of seeding. After 72 h of treatments, the viability was measured using CellTiter-Glo Luminescent Cell Viability Assay Kit (Promega; Madison, WI) as per the manufacturer’s instructions.

### Cell Viability Assays.

Cell viability was determined by MTT assays, as described previously (11). Briefly, 2,000 cells/well were seeded in a 96-well plate 12 h before the indicated treatment. The medium was aspirated, cells washed with 1× PBS, and treated with the indicated chemical agents for 72 h in low pH medium. At the end of the treatment, 50% v/v of 1-mg/mL solution of 3-[4,5-dimethylthiazol-2-yl]-2,5-diphenyltetrazolium bromide (MTT) agent was added for 2 h. The medium containing MTT was aspirated, and the MTT crystals within the cells were dissolved in 100 µL DMSO. Relative cytotoxicity was determined by measuring the absorbance at 570 nm using a Cytation3 plate reader (BioTek Instruments).

### Celigo-Based Proliferation Analysis.

Cells were seeded and treated the same way as for cell viability assays in transparent 96-well plates. Cell confluence was determined by bright-field imaging using the Nexcelom Celigo Imaging Cytometer.

### Clonogenic Assay.

Cell growth and colony formation assay were determined by crystal violet staining. Briefly, cells were seeded in a six-well cell culture plate at a density of 4,000 cells/well clonogenic assay in triplicates. The medium was exchanged every third day post cells seeding. Once a visible number of clones grew, the medium was removed, and the cells were fixed with methanol at 4 °C for 10 min. Subsequently, the cells were stained with crystal violet (0.4%) at room temperature for 20 min. After staining, cells were washed with distilled water and air-dried for observation. The number of colonies was counted manually.

### LC-MS/MS for Polar Metabolites.

Relative quantification of polar metabolites was performed using selected reaction monitoring (SRM)-based mass spectrometry method (43). The cells were washed with cold LC-MS-grade water, and polar metabolites were extracted using 80% LC MS-grade methanol. An ACQUITY UPLC-based BEH amide column (150 mm × 2.1 mm, Waters) was used to separate the metabolites with a gradient buffer composition consisting of Buffer A (100% acetonitrile) and Buffer B (20 mM ammonium acetate, pH 9.0). Polyamines were analyzed by reverse-phase, ion-paired UPLC and were normalized to DNA content. UPLC was performed with an ACQUITY UPLC system (Waters Inc.), using a Waters BEH Amide column (1.7 µm, 2.1 mm × 150 mm). Mobile phase A: acetonitrile. Mobile phase B: water with 20 mM ammonium formate, pH = 4.0. The flow rate was maintained at 0.3 mL/min. Datasets were processed using Skyline (Version 23.1.0.268 MacCoss Lab Software). Metaboanalyst 6.0 webtool was utilized to generate PCA and heatmap visualizations of the resulting datasets. Relative metabolite levels were normalized to the average peak area of the control group and were compared with experimental test groups using one-way ANOVA with Bonferroni’s posttest correction for multiple comparisons. [U-^13^C]glucose-based flux analysis of metabolites was performed as described previously (11).

### shRNA Knockdown or Overexpression.

Lentiviral constructs with short hairpins targeting human *MUC1*, *SAT1,* and scrambled control (shScr) were utilized for generating knockdowns as described previously (25). A pLKO.1 lentiviral expression vector containing the puromycin resistance gene was used to coexpress individual shRNAs. Two hairpin sequences of each gene were selected that reduced the protein levels by >70. *HIF1A* sgRNA constructs for knockouts were purchased from Cellecta. Overexpression of *SAT1 (Homo sapiens)* was achieved by utilizing SAT1 cDNA in pLX304 (DNASU, #HsCD00445109). Recombinant lentiviral particles were produced by transient transfection of plasmids into HEK293T cells. The expression of *MUC1*, *SAT1*, and *HIF1A* were determined by quantitative RT-PCR and western blot.

### RNA Isolation and Real-Time PCR Analysis.

Total RNA was isolated from cells using TRIzol reagent (Thermo Fisher Scientific, 15596026) following the manufacturer’s instructions. Total RNA was quantified, and 2,000 ng of RNA was reverse transcribed to cDNA using an ABI High-Capacity cDNA RT Kit (Thermo Fisher Scientific, 4374966). The cDNA was analyzed by qRT-PCR to evaluate the expression of targeted genes using the SYBR Green PCR Master Mix (Applied Biosystems). The expression of each gene was normalized to the human *RPS18*. The primer pairs used in the study are listed in *SI Appendix*, Table S1. The data were analyzed using the ΔΔCt method, as previously described (44).

### Protein Extraction and Immunoblotting.

Total cellular protein was extracted using RIPA buffer (50 mM Tris-HCl pH 8.0 containing 1% NP-40, 150 mM NaCl, 5 mM EDTA and 1 mM phenylmethylsulfonyl fluoride) containing protease and phosphatase inhibitors. We utilized the following primary antibodies: Anti-HIF-1α (BD Biosciences 610958), Anti-SAT1 (Cell Signaling Technology, 61586), anti-MUC1 (CT2 clone), anti-PGC-1α (Cell Signaling Technology, 2178), Anti-β-actin (DHSB, JLA20), and antivinculin (Sigma Aldrich, V4505). Protein expression levels were normalized to the respective loading control, β-actin or vinculin as indicated.

### XFe96 OCR Analysis.

The OCR and ECAR were analyzed with the Seahorse XF Cell Mito Stress Test and Glyco Stress Test, respectively, in a Seahorse XFe96 Analyzer (Agilent Technologies) as per the manufacturer’s instructions. The experimental design was set up using the WAVE software, and measurements were performed in the Seahorse XFe96 Analyzer. Postmeasurement, the estimated protein contents were used for the normalization of the seahorse data.

### ChIP-qPCR.

ChIP assay was carried out using the Pierce agarose ChIP kit as per the manufacturer’s instructions. In brief, cells were cultured under hypoxia for 6 h. Fragmented chromatin lysate was subjected to immunoprecipitation with an anti- HIF-1α antibody (clone D2U3T, Cell Signaling Technology) followed by quantitative PCR with primer sets specific to the prospective HRE of the *SAT1* promoter. The qPCR data were analyzed using the relative quantification normalized against the untreated cells immunoprecipitated with IgG. The delta Ct (ΔCt) for the difference between Ct values for the antibody of interest (HIF-1α) and the control antibody (IgG) was calculated. The primer pairs used in the study are listed in *SI Appendix*, Table S2. The fold enrichment was determined using the formula 2^-(ΔCt).

### Mitotracker Staining and Imaging.

To image mitochondria, 2 × 10^4^ PDAC cells were seeded per well in 96-well plates. After 24 h, mitochondria were visualized using MitoTracker Green (200 nM) (Cell Signaling Technology, catalog number: 9074) for 20 min at 37 °C. Subsequently, cells were washed three times in 1× PBS buffer. Live images of the cells were captured using the Keyence fluorescence microscope (Keyence Corporation of America, Itasca, IL) and analyzed using ImageJ software.

### Immunohistochemistry and Immunofluorescence.

Tumor tissues were fixed in 10% neutral buffered formalin shortly after harvesting. After 24 to 48 h, fixed tissues were transferred to 70% ethanol. The tissues were embedded in paraffin, and 5 μm sections were mounted on slides. For immunohistochemical analysis of Ki-67, and cleaved caspase-3, antigen retrieval was performed by boiling in citrate buffer (pH 6.0). Slides were incubated overnight at 4 °C with primary antibodies against Ki67 (1:200), and cleaved caspase-3 (1:200). We utilized the following primary antibodies: Anti-Ki-67 (Cell Signaling Technology, 12202), and anti-cleaved caspase-3 (Cell Signaling, 9664S). The stained sections were imaged and captured at 200 × magnification using a Leica DMI6000B Inverted microscope. Cells with Ki-67 positive nuclear staining were counted from three random fields of a section, and a minimum of three tumor sections per group were analyzed.

### Organoid Culture and Assays.

The organoid lines were generated as published previously (45). In brief, PDX tissues were minced and incubated in digestion media (containing 1 mg/mL Collagenase XI, 10 μg/mL DNAse I, 10.5 μmol/L Y-27632 in Human Complete Medium) at 37 °C with mild agitation for up to 1 h. The dissociated cells were collected, plated with Matrigel, and cultured in Human Complete Feeding Medium, which consisted of advanced DMEM/F12, HEPES 10 mmol/L, Glutamax 1×, A83-01 500 nmol/L, hEGF 50 ng/mL, mNoggin 100 ng/mL, hFGF10 100 ng/mL, hGastrin I 0.01 μmol/L, N-acetylcysteine 1.25 mmol/L, nicotinamide 10 mmol/L, PGE2 1 μmol/L, B27 supplement 1× final, R-spondin1 conditioned media 10% final, and Wnt3A-conditioned media 50% final. After 4 d in culture, the organoids were treated with FOLFIRINOX and/or pentamidine and 48 h later cell viability was assessed using CellTiter-Glow assay. The FOLFIRINOX components at 100% dose were Oxaliplatin (250 μM), 5FU (250 μM), Folinic Acid (250 μM) and Irinotecan (25 μM). We used pentamidine at 0.5 μM and 1 μM doses.

### Mouse Strains.

Congenitally athymic nude mice (NCr-nu/nu) (6 to 8 wk age) were bred in-house or purchased from Taconic Biosciences. Mice were housed under pathogen-free conditions. All animal experiments were performed with the approval from the University of Nebraska Medical Center Institutional Animal Care and Use Committee.

### Tumor Growth Studies and Chemotherapy.

Athymic nude mice (NCr-nu/nu) were anesthetized by intraperitoneal injection of ketamine/xylazine (0.1 mg/10 g body weight). To assess the effects of pentamidine and FOLFIRINOX, we orthotopically implanted 0.1 × 10^5^ cancer cells coimplanted with 0.1 × 10^5^ HPS cells in the pancreas of athymic nude mice. Ten days postimplantation, the mice were treated with pentamidine alone (10 mg/kg, every day through intraperitoneal injection), FOLFIRINOX alone, or a combination of pentamidine and FOLFIRINOX or saline as a vehicle control. As additional groups to the same experiment, we orthotopically implanted S2-013 cancer cells knocked down for SAT1 (sh*SAT1-*A) and treated them with FOLFIRINOX. Mice were monitored for 27 d postimplantation, and tumor volumes were measured manually using Vernier calipers. The tumor volumes were calculated by the formula V = 1/2 (Length × Width^2^). Mice were monitored for 27 d and the experiment was terminated when the tumor size reached 10 mm in any cohort.

### Statistical Analyses.

All the experimental assays were performed at least in triplicates, and the values are represented as averages. Error bars indicate the SEM. The most representative experiment of at least three independent repetitions has been reported. The statistical significance was estimated by unpaired two-tailed Student’s *t* test or one-way ANOVA with Bonferroni’s multiple comparisons test. All data analyses were conducted using GraphPad Prism software (version 5, version 8). The *P*-value significance of 0.01 < *P* < 0.05, 0.001 < *P* < 0.01, and *P* < 0.001 were represented by *, **, and *** respectively, as shown in figures and tables.

## Supplementary Material

Appendix 01 (PDF)

## Data Availability

All study data are included in the article and/or *SI Appendix*. Sequencing data for control and *SAT1* knockdown S2-013 cells is available through SRA, NCBI under accession code PRJNA1086935 (46).
