# Supplementary material for: The MUC1–HIF-1α signaling axis regulates pancreatic cancer pathogenesis through polyamine metabolism remodeling
Source: Proc Natl Acad Sci U S A. 2024 Mar 28;121(14):e2315509121. doi: 10.1073/pnas.2315509121 (PMC10998584; doi:10.1073/pnas.2315509121)
Supplement: Supplementary file 1 — Appendix 01 (PDF) [file pnas.2315509121.sapp.pdf]

SUPPLEMENTARY FIGURES AND LEGENDS

Figure S1

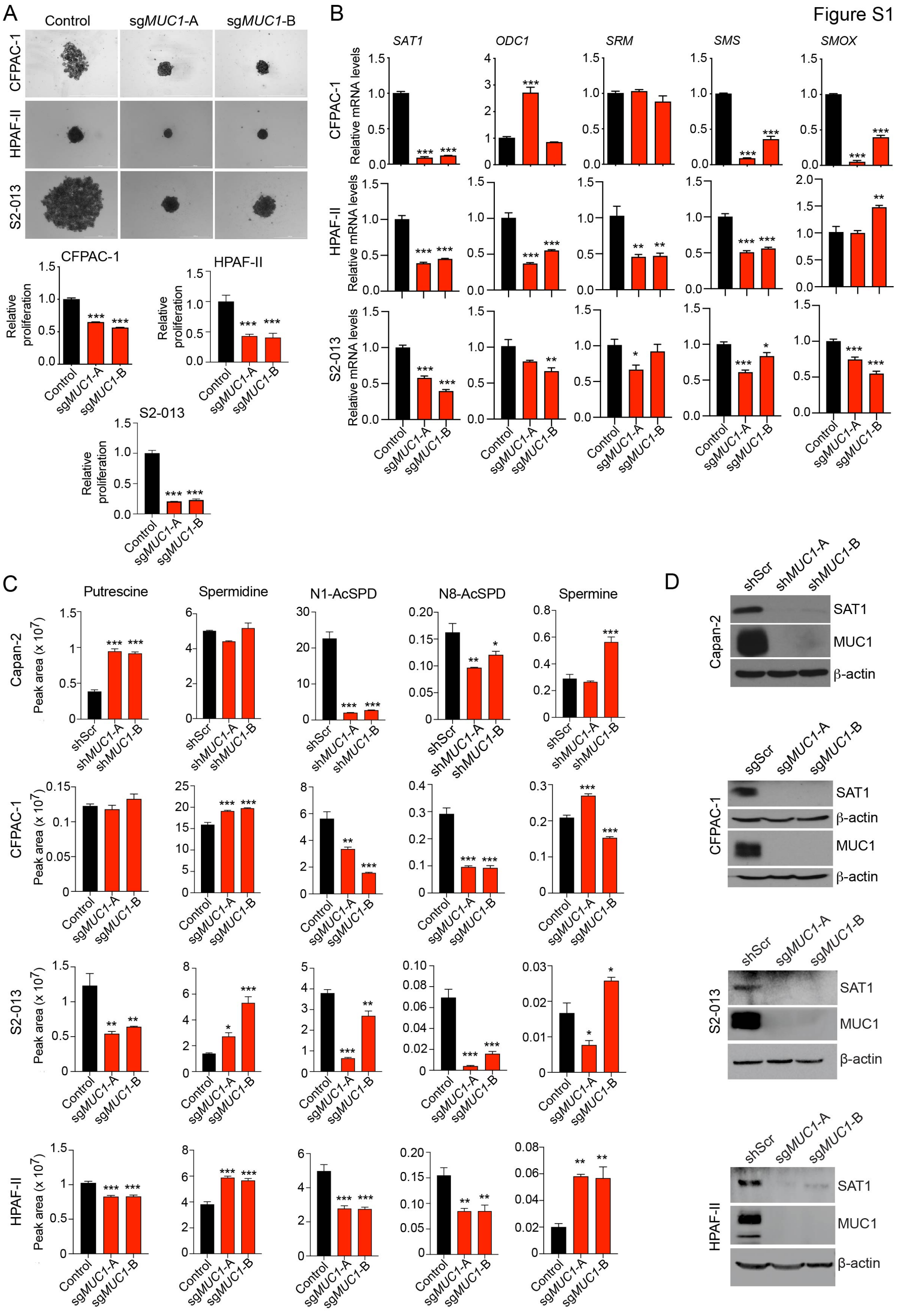
A
Control
sgMUC1-A
sgMUC1-B
CFPAC-1
HPAF-II
S2-013
Relative proliferation
B
SAT1
ODC1
SRM
SMS
SMOX
Relative mRNA levels
C
Putrescine
Spermidine
N1-AcSPD
N8-AcSPD
Spermine
Capan-2
Peak area (x 10^7)
shScr
shMUC1-A
shMUC1-B
D
SAT1
MUC1
β-actin

Figure S1. MUC1 regulates SAT1 in pancreatic cancer cells.

(A) 3D spheroid growth analysis of control and *MUC1* knockout CFPAC-1, HPAF-II, and S2-013 cells along with relative quantitation. Scale bar: 1000 μm.

(B) Relative mRNA expression of *MUC1* and polyamine pathway genes (*SAT1*, *ODC1*, *SRM*, *SMS*, and *SMOX*) in control and *MUC1* knockout CFPAC-1, HPAF-II, and S2-013 cells.

(C) The relative levels of polyamine metabolites in control and *MUC1* knockdown Capan-2 and *MUC1* knockout CFPAC-1, S2-013, and HPAF-II cells.

(D) MUC1 and SAT1 protein levels in control and *MUC1* knockdown Capan-2 and *MUC1* knockout CFPAC-1, S2-013, and HPAF-II cells by immunoblotting.

The bar charts are represented as mean ± SEM and compared by one-way ANOVA with Tukey's post-hoc test (A, B, and C). $*p < 0.05$, $**p < 0.01$, and $***p < 0.001$

Figure S2

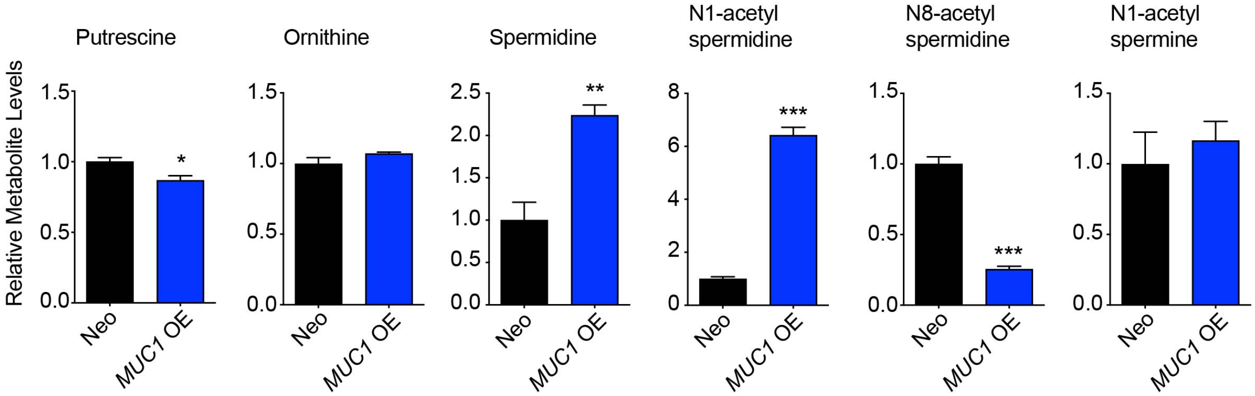

Figure S2. MUC1 regulates polyamine metabolites in the pancreatic cancer cells.

The relative levels of polyamine pathway metabolites in *MUC1* overexpressing S2-013 cells relative to control cells as determined by LC-MS/MS-based metabolomics.

The data is represented as mean ± SEM and compared by Student's t-test. $*p < 0.05$, $**p < 0.01$, and $***p < 0.001$

Figure S3

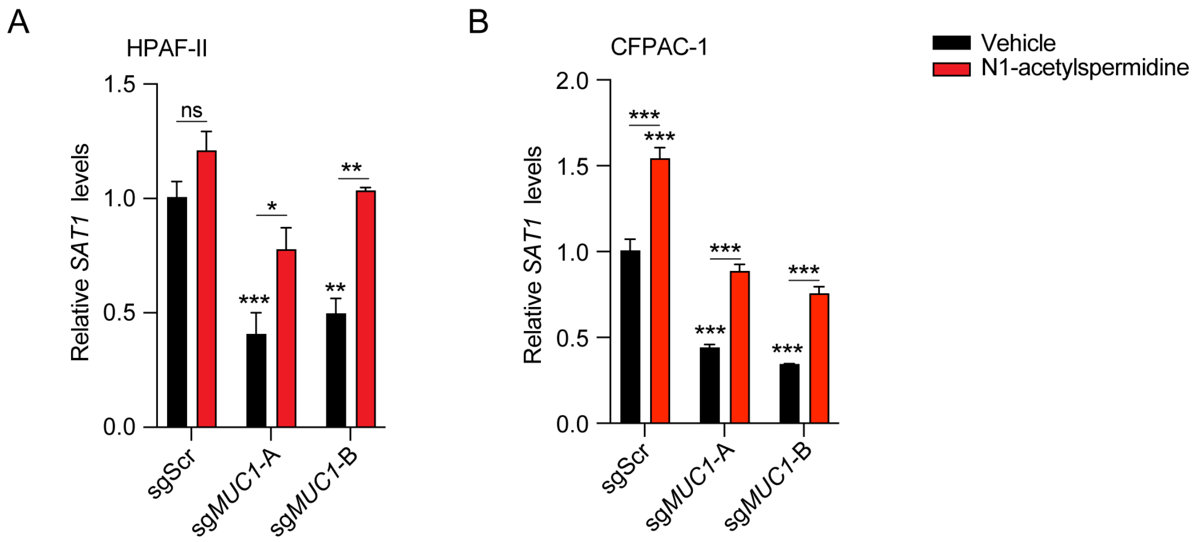

Figure S3. Exogenous addition of N1-acetylspermidine regulates SAT1 gene expression.

(A-B) Relative mRNA expression of *SAT1* gene in scrambled control and *MUC1* knockout HPAF-II (A) and CFPAC-1 (B) cells upon treatment with N1-acetylspermidine (100 μM) and cultured for 24 hr under hypoxic conditions.

Bar charts are represented as mean ± SEM and compared by unpaired Student's t-test (A) and one-way ANOVA with Tukey's post-hoc test (B, C). $*p < 0.05$, $**p < 0.01$, and $***p < 0.001$

Figure S4

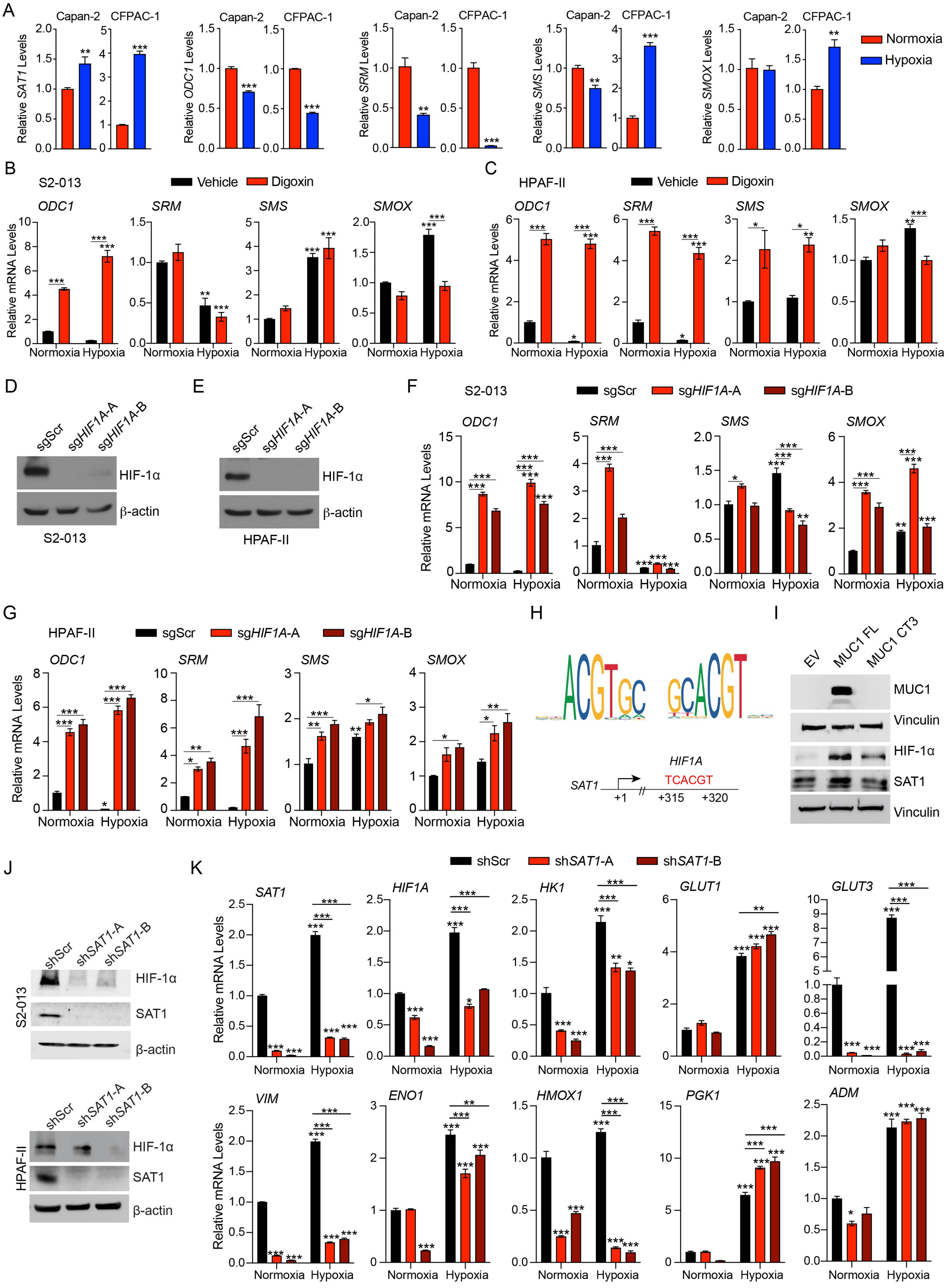
A
Capan-2
CFPAC-1
Relative SAT1 Levels
Relative ODC1 Levels
Relative SRM Levels
Relative SMS Levels
Relative SMOX Levels
Normoxia
Hypoxia
B
S2-013
Vehicle
Digoxin
ODC1
SRM
SMS
SMOX
Relative mRNA Levels
C
HPAF-II
D
sgScr
sgHIF1A-A
sgHIF1A-B
HIF-1α
β-actin
S2-013
E
HPAF-II
F
G
H
HIF1A
TCACGT
SAT1
+1
+315
+320
I
EV
MUC1 FL
MUC1 CT3
MUC1
Vinculin
SAT1
J
shScr
shSAT1-A
shSAT1-B
K
HIF1A
HK1
GLUT1
GLUT3
VIM
ENO1
HMOX1
PGK1
ADM

Figure S4. Effect of HIF-1α inhibition/deletion on the expression of polyamine pathway genes.

(A) Relative mRNA expression of *SAT1*, *ODC1, SRM, SMS,* and *SMOX* genes in Capan-2 and CFPAC-1 cells cultured under normoxia and hypoxia.

(B, C) Relative mRNA expression of *ODC1, SRM, SMS,* and *SMOX* genes in S2-013 (B) and HPAF-II (C) cells cultured under normoxia and hypoxia upon digoxin treatment for 24 hours.

(D, E) HIF-1α protein levels in control and *HIF1A* knockout S2-013 (D) and HPAF-II (E) cells culture under hypoxia for 6 hours.

(F, G) Relative mRNA expression of *ODC1, SRM, SMS,* and *SMOX* genes in control and *HIF1A* knockout S2-013 (F) and HPAF-II (G) cells cultured under normoxia or hypoxia.

(H) The consensus sequence of HIF-1α binding motif (top) and the putative HIF-1α binding site in *SAT1* promoter region (bottom).

(I) MUC1, HIF-1α, and SAT1 protein levels in S2-013 cells over-expressing empty vector (EV), FLAG-tagged full-length MUC1 (FL) or cytoplasmic tail-deleted MUC1 (CT3) protein under hypoxia.

(J) HIF-1α and SAT1 protein levels in scramble control and *SAT1* knockdown S2-013 and HPAF-II cells culture under hypoxia.

(K) Relative mRNA expression of *SAT1*, *HIF1A* and HIF-1α regulated genes (*HK1*, *GLUT1*, *GLUT3*, *VIM*, *ENO1*, *HMOX1*, *PGK1*, and *ADM*) in scramble control and *SAT1* knockdown S2-013 cultured under normoxic and hypoxic conditions.

Bar charts are represented as mean ± SEM and compared by unpaired Student's t-test (A) and one-way ANOVA with Tukey's post-hoc test (B, C, F, G, and K). $*p < 0.05$, $**p < 0.01$, and $***p < 0.001$

Figure S5

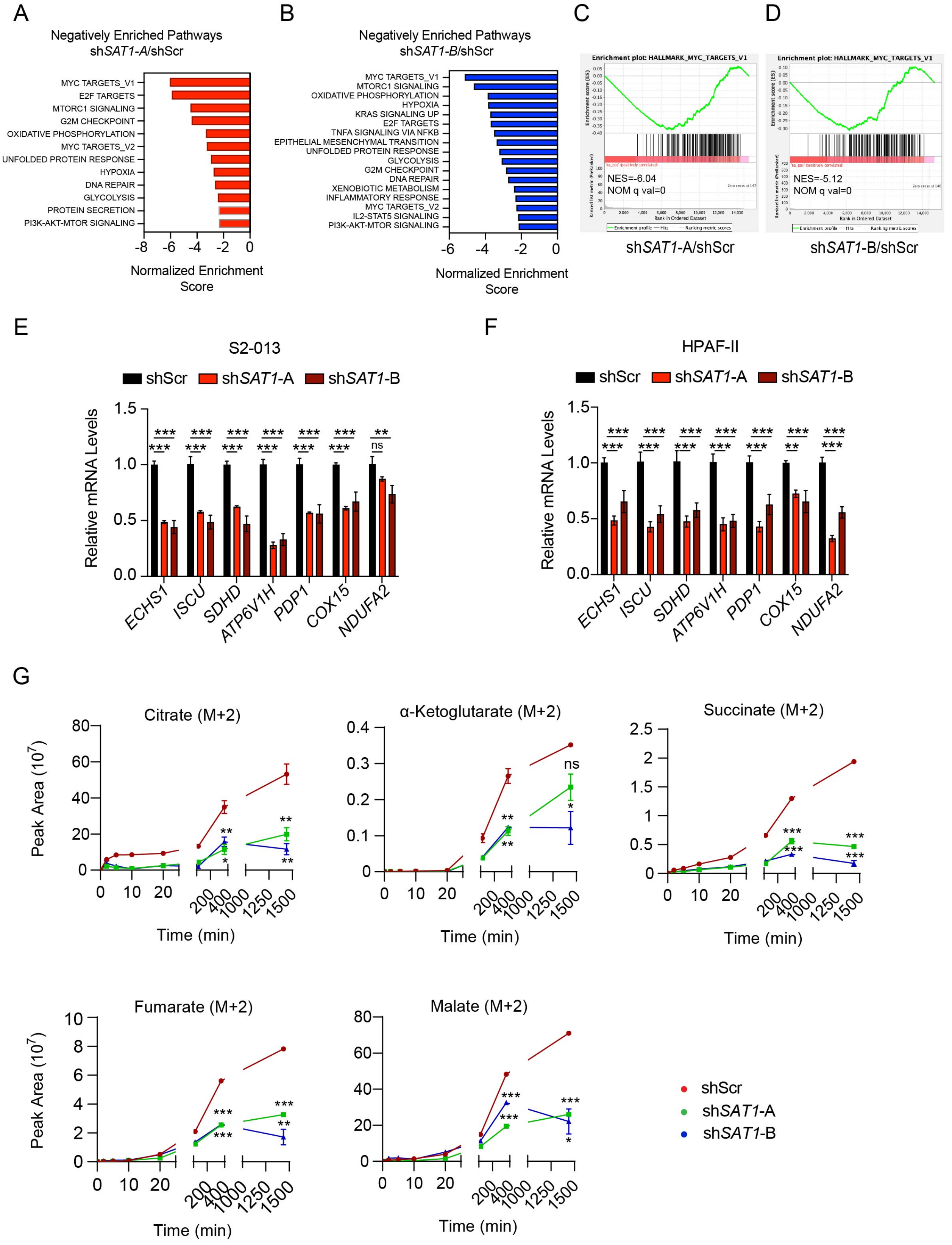
A
Negatively Enriched Pathways
shSAT1-A/shScr
MYC TARGETS_V1
E2F TARGETS
MTORC1 SIGNALING
G2M CHECKPOINT
OXIDATIVE PHOSPHORYLATION
MYC TARGETS_V2
UNFOLDED PROTEIN RESPONSE
HYPOXIA
DNA REPAIR
GLYCOLYSIS
PROTEIN SECRETION
PI3K-AKT-MTOR SIGNALING
Normalized Enrichment Score
B
Negatively Enriched Pathways
shSAT1-B/shScr
MYC TARGETS_V1
MTORC1 SIGNALING
OXIDATIVE PHOSPHORYLATION
HYPOXIA
KRAS SIGNALING UP
E2F TARGETS
TNFA SIGNALING VIA NFKB
EPITHELIAL MESENCHYMAL TRANSITION
UNFOLDED PROTEIN RESPONSE
GLYCOLYSIS
G2M CHECKPOINT
DNA REPAIR
XENOBIOTIC METABOLISM
INFLAMMATORY RESPONSE
MYC TARGETS_V2
IL2-STAT5 SIGNALING
PI3K-AKT-MTOR SIGNALING
Normalized Enrichment Score
C
Enrichment plot: HALLMARK_MYC_TARGETS_V1
NES=-6.04
NOM q val=0
shSAT1-A/shScr
D
Enrichment plot: HALLMARK_MYC_TARGETS_V1
NES=-5.12
NOM q val=0
shSAT1-B/shScr
E
S2-013
shScr shSAT1-A shSAT1-B
Relative mRNA Levels
ECHS1 ISCU SDHD ATP6V1H PDP1 COX15 NDUFA2
F
HPAF-II
shScr shSAT1-A shSAT1-B
Relative mRNA Levels
ECHS1 ISCU SDHD ATP6V1H PDP1 COX15 NDUFA2
G
Citrate (M+2)
α-Ketoglutarate (M+2)
Succinate (M+2)
Fumarate (M+2)
Malate (M+2)
Peak Area (10^7)
Time (min)
shScr
shSAT1-A
shSAT1-B

Figure S5. *SAT1* knockdown alters oxidative phosphorylation of pancreatic cancer cells.

(A, B) Bar plots of negatively enriched pathways from GSEA analysis of sh*SAT1*-A (A) and sh*SAT1*-B (B) cells compared to shScr S2-013 cells. The pathways with NES score greater than 2 and q-value less than 0.05 are shown.

(C, D) GSEA plot of MYC targets-V1 pathway genes in sh*SAT1*-A (C) and sh*SAT1*-B (D) clones compared to shScr S2-013 cells.

(E, F) Relative mRNA expression of mitochondrial genes in scramble control and *SAT1* knockdown S2-013 (E) and HPAF-II (F) cells.

(G) Kinetics of incorporation of ^{13}C label from [U-^{13}C]glucose into TCA metabolites in shScr and sh*SAT1* HPAF-II cell line, as identified by LC-MS/MS analysis. M+X represents the number of ^{13}C labeled carbon atoms in each metabolite, presented in arbitrary peak intensity units.

The data is represented as mean ± SEM, and the statistical significance is calculated by one-way ANOVA with Tukey's post-hoc test (E and F). $*p < 0.05$, $**p < 0.01$, and $***p < 0.001$

Figure S6

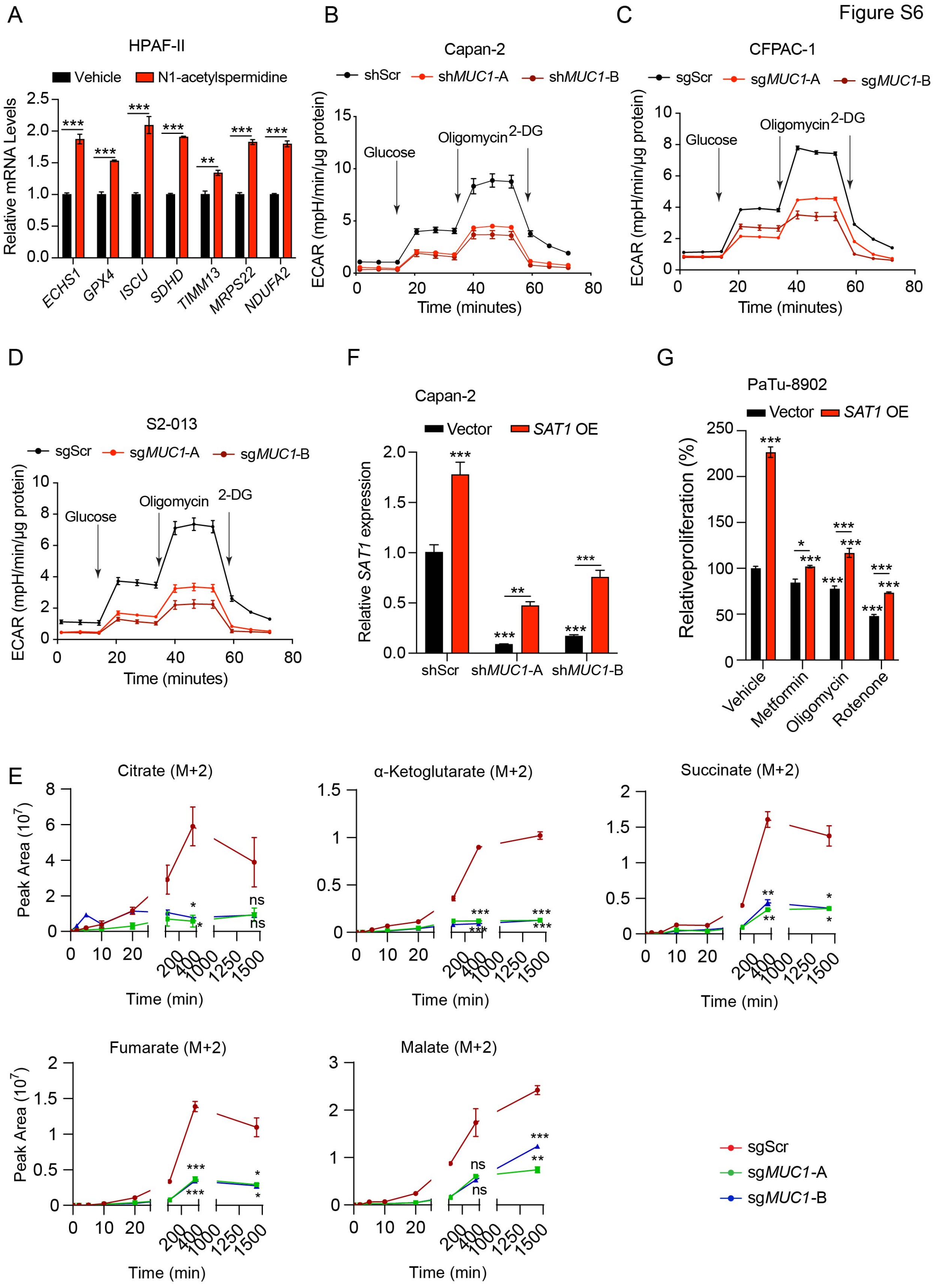
A
HPAF-II
Vehicle
N1-acetylspermidine
Relative mRNA Levels
ECHS1
GPX4
ISCU
SDHD
TIMM13
MRPS22
NDUFA2
B
Capan-2
shScr
shMUC1-A
shMUC1-B
ECAR (mpH/min/µg protein)
Glucose
Oligomycin
2-DG
Time (minutes)
C
CFPAC-1
sgScr
sgMUC1-A
sgMUC1-B
D
S2-013
F
Capan-2
Vector
SAT1 OE
Relative SAT1 expression
shScr
shMUC1-A
shMUC1-B
G
PaTu-8902
Vector
SAT1 OE
Relativeproliferation (%)
Vehicle
Metformin
Oligomycin
Rotenone
E
Citrate (M+2)
α-Ketoglutarate (M+2)
Succinate (M+2)
Fumarate (M+2)
Malate (M+2)
Peak Area (10^7)
Time (min)
sgScr
sgMUC1-A
sgMUC1-B

Figure S6. MUC1-SAT1 axis regulates energy metabolism of pancreatic cancer cells.

(A) Relative mRNA expression of mitochondrial genes upon treatment with N1-acetylspermidine in HPAF-II cells.

(B, C, D) Seahorse-based metabolic flux analysis of extracellular acidification rates (ECAR) in scramble control and *MUC1* knockdown Capan-2 (B), *MUC1* knockout CFPAC-1 (C), and *MUC1* knockout S2-013 (D) cells.

(E) Kinetics of incorporation of ^{13}C label from [U-^{13}C]glucose into TCA metabolites in sgScr and sg*MUC1* S2-013 cell line, as identified by LC-MS/MS analysis. M+X represents the number of ^{13}C labeled carbon atoms in each metabolite, presented in arbitrary peak intensity units.

(F) Relative *SAT1* mRNA levels in *MUC1* knockdown Capan-2 vector control and *SAT1* overexpressing cells compared to the scrambled control cells.

(G) Relative proliferation of vector control and *SAT1* overexpressing PaTu-8902 cells upon treatment with OXPHOS inhibitors (5 mM metformin, 3 nM oligomycin, and 10 μM rotenone). Each group has been normalized to vehicle vector control. Note: this is the same data as in Figure 6L but plotted differently (normalized to vehicle vector control) to visualize the effect of *SAT1* overexpression on cell growth.

Bar charts are represented as mean ± SEM and compared by unpaired Student's t-test (A) and one-way ANOVA with Tukey's post-hoc test (F & G). $*p < 0.05$, $**p < 0.01$, and $***p < 0.001$

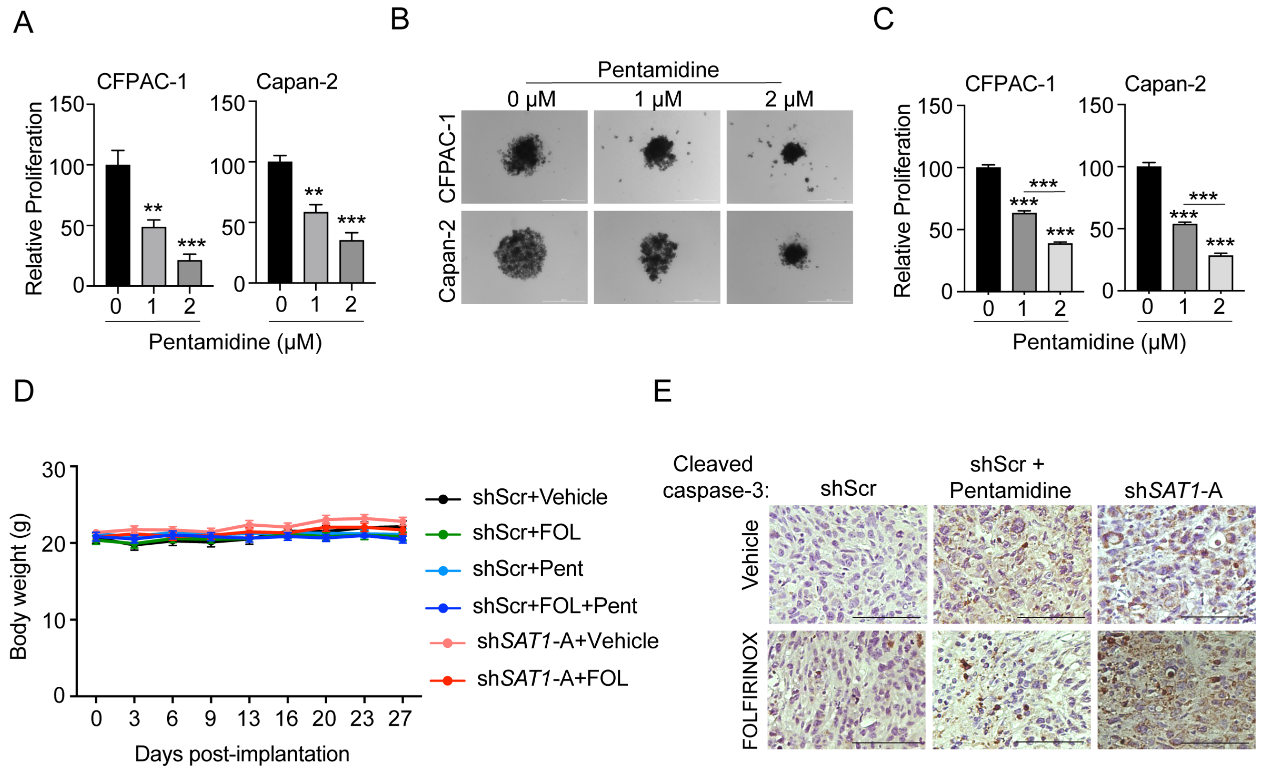

Figure S7. Pentamidine and FOLFIRINOX combination treatment does not affect the body weight of PDAC tumor-bearing mice.

(A) Relative proliferation of CFPAC-1 and Capan-2 cells upon treatment with pentamidine for 72 hrs.

(B-C) The representative images (B) and quantitation (C) of 3D spheroid growth assay in CFPAC-1 and Capan-2 cells upon treatment with pentamidine for 72 hours.

(D) Body weight measurements over the course of the experiment in athymic nude mice implanted with shScr or sh*SAT1*-A S2-013 cells upon treatment with FOLFIRINOX, without and with pentamidine.

(E) Representative IHC staining for cleaved caspase-3 in formalin-fixed tumor sections from athymic nude mice implanted with shScr or sh*SAT1*-A S2-013 cells upon treatment with FOLFIRINOX, without and with pentamidine. Scale bar = 100 μm.

The data is represented as mean ± SEM and compared by one-way ANOVA with Tukey's post-hoc test (A, C, and D). $*p < 0.05$, $**p < 0.01$, and $***p < 0.001$

Supplementary Table 1.

Gene name	Forward primer sequence	Reverse primer sequence
MUC1	TGCCGCCGAAAGAACTACG	TGGGGTACTCGCTCATAGGAT
SAT1	ACCCGTGGATTGGCAAGTTAT	TGCAACCTGGCTTAGATTCTTC
ODC1	TTTACTGCCAAGGACATTCTGG	GGAGAGCTTTTAACCACCTCAG
SRM	GTGGTGGCCTATGCCTACTG	CTCCTGGAAGTTCGTGCTCG
SMS	TGGGCGGGTGAAACGATTAC	CCAAACTGCTTCGAGTGTAGAA
SMOX	AGTTTGTGTGGGAGGACGAA	TGCCTCGTCATCACACTTCT
RPS18	GAGGATGAGGTGGAACGTGT	AGAAGTGACGCAGCCCTCTA
GPX4	GAGGCAAGACCGAAGTAAACTAC	CCGAACTGGTTACACGGGAA
MRPS22	CCCCTCGGAACAACTGTATTG	AAAGAGCAAGGTAGCGGTTGG
NDUFA2	GCAGCAAGTCGAGGAGTCG	CGTTTCTCAATGAAGTCCCTGA
ECHS1	TGTCCTGTTGAGACACTGGTG	ACAAACGCGGTCATCCCTTC
ISCU	GGGTCCCTTGACAAGACATCT	CCTTTCACCCATTCAGTGGCTA
SDHD	TTGCTCTGCGATGGACTATTCC	CAAGGCATCCCCATGAACAT
TIMM13	CAGAGGATGACGGACAAGTGT	CATGTAGCGGTCCATGCACA
ADM	ATGAAGCTGGTTTCCGTCG	GACATCCGCAGTTCCCTCTT
HMOX1	AAGACTGCGTTCCTGCTCAAC	AAAGCCCTACAGCAACTGTCG
GLUT3	GCTGGGCATCGTTGTTGGA	GCACTTTGTAGGATAGCAGGAAG
GLUT1	GATTGGCTCCTTCTCTGTGG	TCAAAGGACTTGCCCAGTTT
PGK1	TTAAAGGGAAGCGGGTCGTTA	TCCATTGTCCAAGCAGAATTTGA
ENO1	CTGGTGCCGTTGAGAAGGG	GGTTGTGGTAAACCTCTGCTC
VIM	GACGCCATCAACACCGAGTT	CTTTGTCGTTGGTTAGCTGGT
HK1	GGAAGCAGACGCACAACAATG	CACGGAAATTGGTTCCTCCAA
PDP1	CCTGCAAATGCACCCATTGAG	CTGCCTGGGAACAAGCACAA
COX15	CAGCGCCTAGAGCACAGTG	GCCAGACTCTGTCAACCTAGT
ATP6V1H	CTTTCTGCCAATGTTGAATCGC	GTCACTGCCTTCCATCAGTTC

Supplementary Table 2.

Primer name	Forward	Reverse
ChIP encompassing HRE element on SAT1 promoter	GCCGACTGCAGTGACATACT	CGCAGAAAGGGGAACAGG
